# Allogeneic stem cell transplantation for major T-cell lymphoma entities: an analysis of the EBMT-lymphoma working party

**DOI:** 10.1186/s13045-026-01783-w

**Published:** 2026-02-21

**Authors:** Evgenii Shumilov, Maud Ngoya, Philipp Berning, Raynier Devillier, Edouard Forcade, Thomas Schroeder, Frank Kroschinsky, Matthias Stelljes, Veronika Valkova, Francesca Kinsella, Patrice Chevallier, Gitte Olesen, Mohamad Mohty, Flore Sicre de Fontbrune, Eva Wagner-Drouet, Robert Zeiser, Marco Herling, Georg-Nikolaus Franke, Lucía López-Corral, Francis Ayuk, Georg Lenz, Gerald Wulf, Anna Sureda, Arain Laurence, Peter Dreger, Ali Bazarbachi, Norbert Schmitz

**Affiliations:** 1 Department of Medicine A, Hematology and Oncology, University Hospital of Muenster, Albert-Schweitzer-Campus 1 A1, 48149 Muenster, Germany; 2 European Society for Blood and Marrow Transplantation, Paris Study Unit, Hôpital Saint-Antoine, Paris, France; 3 Department of Medicine, Lymphoma Service, Memorial Sloan Kettering Cancer Center, New York, USA; 4 Aix-Marseille Univ, MSC Lab, Department of Hematology, Institut Paoli-Calmettes, Marseille, France; 5 CHU Bordeaux, Hôpital Haut-Leveque, Pessac, France; 6 Department of Hematology and Stem Cell Transplantation, West German Cancer Center Essen, University Hospital Essen, Essen, Germany; 7 Department of Internal Medicine I, University Hospital Carl Gustav Carus, TU Dresden, Dresden, Germany; 8 Institute of Hematology and Blood Transfusion, Prague, Czech Republic; 9 Centre for Clinical Haematology, Queen Elizabeth Hospital Birmingham, Birmingham, UK; 10 Hematology Department, CHU Hotel-Dieu, Nantes, France; 11 Department of Hematology, Aarhus University Hospital, Aarhus, Denmark; 12 Hematology Department, Hôpital Saint-Antoine, and Université Pierre & Marie Curie, Paris, France; 13 Hôpital Saint Louis, AP-HP, Unité d’Hématologie Et Transplantation, Paris, France; 14 Department of Medicine, Haematopoietic Stem Cell Transplantation, University Medical Center Mainz, Mainz, Germany; 15 Department of Hematology, Oncology and Stem Cell Transplantation, Faculty of Medicine, Freiburg University Medical Center, Freiburg, Germany; 16 Department for Hematology, Cell Therapy and Hemostaseology, University Hospital Leipzig, Leipzig, Germany; 17 Department of Hematology, IBSAL, CIBERONC, Hospital Universitario de Salamanca, Salamanca, Spain; 18 Department of Stem Cell Transplantation, University Medical Center Hamburg-Eppendorf, Hamburg, Germany; 19 Department of Hematology and Medical Oncology, University Hospital of Goettingen, Goettingen, Germany; 20 Clinical Hematology Department, Institut Català d’Oncologia - L’Hospitalet, IDIBELL, Universitat de Barcelona, Barcelona, Spain; 21 Department of Haematology, University College London Hospital, London, UK; 22 Department of Hematology & Oncology, University Hospital Heidelberg, Heidelberg, Germany; 23 Bone Marrow Transplantation Program, Department of Internal Medicine, American University of Beirut, Beirut, Lebanon

**Keywords:** PTCL NOS, AITL, ALK-negative ALCL, Allogeneic stem cell transplantation, EBMT

## Abstract

**Background:**

Allogeneic hematopoietic stem cell transplantation (allo-SCT) is an established treatment for peripheral T-cell lymphoma (PTCL), particularly for patients with relapsed/refractory (r/r) disease. We aimed to retrieve novel information on the role of histology, disease status prior to transplantation, and donor choice for patients with PTCL not otherwise specified (NOS), angioimmunoblastic T-cell lymphoma (AITL), and anaplastic lymphoma kinase (ALK)-negative ALCL. We compared imaging by computed tomography (CT) or positron emission tomography (PET) for defining disease status prior to allo-SCT.

**Methods:**

Eligible were adult patients with PTCL-NOS, AITL, and ALK-negative ALCL undergoing allo-SCT between 2010 and 2022 and reported to EBMT.

**Results:**

1958 patients underwent allo-SCT. Of patients with known number of prior lines of therapies (n = 1310), 301 (23%), 431 (32.9%) and 578 (44.1%) patients received allo-SCT after one (1L), two (2L) or three or more therapy lines (3L +), respective. Three-year GvHD-free, relapse-free survival (GRFS), progression-free survival (PFS) and overall survival (OS) were 35.8%, 50.9% and 56.8%, respectively. Three-year relapse incidence (RI) and non-relapse mortality were 25.1% and 24.1%, respectively. In multivariate analysis, histology other than AITL, no complete response (CR) at transplantation, having a haploidentical donor and higher age at allo-SCT resulted in significantly lower PFS and/or OS. Prior autologous SCT had no impact on the results of allo-SCT and major outcomes did not significantly change when the analyses were restricted to the patients with PET-based response at allo-SCT. Patients allografted in partial response (PR) or SD/PD still achieved long-term survival with a 3-year PFS/OS of 46%/53.7% and 39.6%/43.6%, respectively.

**Conclusion:**

Allo-SCT is a valid treatment option in relapsed/refractory PTCL where targeted therapies still play a limited role. Patients with AITL survived significantly better than patients with PTCL NOS or ALK-negative ALCL following a significantly lower RI, also when comparing CR/complete metabolic response (CMR) and PR patients separately. Higher age and non-CR at allo-SCT are associated with worse outcomes.

**Supplementary Information:**

The online version contains supplementary material available at 10.1186/s13045-026-01783-w.

## Background

T-cell lymphomas comprise a heterogeneous group of malignancies derived from mature T-cells accounting for around 10% of all lymphomas diagnosed in the Western world [1]. The most frequent non-cutaneous, non-leukemic T-cell lymphoma entities are peripheral T-cell lymphoma not otherwise specified (PTCL NOS), angioimmunoblastic T-cell lymphoma (AITL), anaplastic large cell lymphoma (ALCL), anaplastic lymphoma kinase (ALK)-negative, and ALCL, ALK-positive. While patients with ALK-positive ALCL carry a significantly better prognosis [2], the other entities mostly show an aggressive clinical course with 5-year overall survival (OS) rates between 30–50% [3–5].

Relapsed/refractory (r/r) T-cell lymphoma remains particularly challenging to treat with a median OS of less than 6 months [6–8]. Although multiple drugs and modalities such as histone deacetylase inhibitors, pathway inhibitors, antibody drug conjugates (ADC), checkpoint inhibitors, and chimeric antigen receptor -T (CAR-T) cells have been investigated, only brentuximab vedotin (BV) targeting the CD30 antigen frequently present on ALCL cells found its way into clinical routine and international guidelines [9, 10].

The graft-versus lymphoma (GvL) effect exerted by allogeneic stem cell transplantation (allo-SCT) seems particularly strong in T-cell lymphoma [11, 12] making allo-SCT the preferred modality to treat transplant-eligible patients with r/r T-cell lymphoma.

Due to numerous improvements including better donor selection, conditioning, graft-versus-host disease (GvHD) prophylaxis, and supportive care, the mortality and morbidity associated with allo-SCT considerably decreased over the past decades, allowing transplants to be offered also to older and comorbid patients with PTCL.

We performed a detailed analysis of large numbers of patients evaluating recent outcomes of allo-SCT for major T-cell lymphoma entities as reported to the European Society for Blood and Marrow Transplantation (EBMT) registry.

## Methods

### Study design and data collection

This is a retrospective registry-based, multi-center study. Data were provided by the Lymphoma Working Party (LWP) of the EBMT. EBMT is a voluntary group of transplant centers requiring to report all consecutive SCTs and follow-ups once a year. All participating institutions are required to obtain written informed consent from patients prior to registration with the EBMT, following the Helsinki Declaration guidelines. We included adult patients (≥ 18 years) transplanted with PTCL NOS, AITL, and ALK-negative ALCL between January 2010 and December 2022 who had received allo-SCT either up-front or in r/r disease as first SCT or after a preceding autologous SCT (auto-SCT). We retained the diagnosis of AITL, as re-naming of the entity to follicular helper T-cell lymphoma with AITL remaining the most prevalent subgroup was introduced in 2022 only. Patients with ALK-positive ALCL were not included in this analysis because these patients show a significantly better prognosis than other major entities even before BV was introduced and their treatment before transplantation is different with first-line therapy often including etoposide [2], BV [9] or both. Baseline and transplantation characteristics as well as outcomes of eligible patients were retrieved from the EBMT registry. The present study has been approved by the EBMT Lymphoma Working Party, and all accredited EBMT centers obtained informed consent before data registration with EBMT, in accordance with the Helsinki Declaration of 1975.

### Definitions

Diagnosis was based on local pathology reports. Patients were staged according to the Ann-Arbor system. Disease status at transplantation was assessed by local investigators according to standard criteria [(CR (complete remission), PR (partial remission), SD (stable disease) and PD (progressive disease)] and classified as chemosensitive (CR/PR), or chemorefractory disease (SD/PD). While computed tomography (CT) was the standard imaging procedure in earlier years, this was replaced by positron emission tomography (PET) in recent years. PET data were analyzed for a subgroup of patients with available data. Myeloablative conditioning (MAC) was defined as a regimen containing either TBI with a dose greater than 6 Gy, a total dose of oral BU greater than 8 mg/kg, or a total dose of intravenous BU greater than 6.4 mg/kg or melphalan at doses > 140 mg/m^2^. All other regimens are defined as reduced intensity conditioning (RIC) [13]. The diagnosis and grading of acute GvHD (aGVHD) [14] and chronic GVHD (cGvHD) [15] was performed by transplant centers using standard criteria.

### Statistics

Endpoints analyzed were progression-free survival (PFS; survival without lymphoma relapse or progression), OS (time from transplantation to death from any cause), non-relapse mortality (NRM) (death without previous relapse) and relapse incidence (RI) (disease recurrence). GvHD-free, relapse-free survival (GRFS) was calculated using the EBMT definition for registry- based analyses where the time to first event of the following is recorded: severe grade III or IV acute GvHD, severe chronic GvHD, relapse, death. All outcomes were measured from the day of transplantation. Surviving patients were censored at the time of last contact. Probabilities of OS and PFS were calculated using the Kaplan–Meier method. Cumulative incidences for RI and NRM were calculated using a competing risk model, where death was treated as a competing event. Death and relapse were considered as competing events for calculations of aGvHD and cGvHD. Follow-up was calculated from the time of SCT to death or the last follow-up report. Median follow up was calculated by using the reverse Kaplan Meier method. Demographics were compared between groups using the chi-squared test or Fisher’s exact test for categorical variables and the Mann–Whitney U test for continuous variables. Univariate analyses were performed using the log-rank test for PFS and OS, while Gray’s test was used for competing risk outcome data. Multivariate analyses were performed using the Cox proportional-hazards regression model. Results are reported as hazard ratios (HR) with a 95% confidence interval (95% CI). All statistical tests were two-sided with a type I error fixed at 0.05 for factors associated with time-to-event outcomes. All analyses were performed using R version 4.3.3 with the R packages survival version 3.5–8, cmprsk version 2.2–11 and Hmisc version 5.1–2. (R Core Team. R: a language for statistical computing. 2014. R Foundation for Statistical Computing, Vienna, Austria).

## Results

### Patient characteristics

The study population consisted of 1958 patients receiving allo-SCT between 2010 and 2022. Major patient- and procedure-related characteristics are shown in Table 1. The population included patients with PTCL-NOS (n = 949; 48.5%), AITL (n = 762; 38.9%), and ALK-negative ALCL (n = 247; 17.7%). Two thirds of the patients were male, their median age was 54 years, and 69% had a Karnofsky performance score (KPS) ≥ 90% at allo-SCT. 23% of patients had received one line (1L), 32.9% two (2L), and 44.1% three or more lines (3L +) of therapy prior to allo-SCT. 43.7% of patients (n = 856) had received a prior auto-SCT. The median time from diagnosis to allo-SCT was 15.5 months. Disease status at the time of allo-SCT was CR in 52.2%, PR in 27.7%, and SD/PD in 20.2%. Metabolic remission status at allo-SCT by PET was available for 814 patients (41.6%) with 458 of them (56.3%) being in complete metabolic remission (CMR). Most patients had an unrelated donor or a matched related donor (53.4% and 33.8% of the cases, respectively). Of note, 250 patients (12.8%) received a haplo-identical graft. Of 241 patients with available information, 221 patients (91.7%) received post-transplant cyclophosphamide (PTCY) for GvHD prophylaxis. RIC or MAC was used in 60.2% and 39.8% of cases, respectively. Four hundred ninety-nine patients (25.6%) had TBI as part of the conditioning. The numbers and the percentage of patients allografted in CR increased from 18.4% in 2010–2012 to 36.9% in 2019–2022. (Supplemental Table S1). The characteristics of patients undergoing allo-SCT after only one line of therapy or being transplanted in SD/PD are presented Supplemental Table S3 and S4.

**Table 1:** Major characteristics of patients undergoing allo-SCT

Variable	Total	PTCL NOS	AITL	ALK-neg. ALCL	p-value
	**n = 1958 (100%)**	**n = 949 (48.5%)**	**n = 762 (38.9%)**	**n = 247 (17.7%)**	
Median age at allo-SCT (range) [IQR]	54.4 (18–78.5) [45.7–60.6]	52.7 (18–78.5) [42.8–59.7]	56.3 (19–75.9) [49.5–62.1]	52.6 (19.9–73.3) [43.2–59.2]	** < 0.0001**
Time from diagnosis to allo-SCT					** < 0.0001**
Median (range) [IQR]	15.5 (2–389.1) [9.8–26.9]	15.2 (2–264) [9.2–27.7]	14.6 (2.7–318.2) [9.8–23.4]	19.3 (3–389.1) [12.9–31.1]	
≤ 12 months	697 (35.6)	366 (38.6)	278 (36.5)	53 (21.5)	-
> 12 months	1260 (64.4)	582 (61.4)	484 (63.5)	194 (78.5)	-
Sex patient					0.14
Female	655 (33.5)	307 (32.4)	274 (36)	74 (30)	
Male	1302 (66.5)	641 (67.6)	488 (64)	173 (70)	
Unknown	1	1	0	0	
Sex donor					0.43
Female	654 (33.9)	307 (32.9)	268 (35.6)	79 (32.2)	
Male	1278 (66.1)	627 (67.1)	485 (64.4)	166 (67.8)	
Unknown	26	15	9	2	
Female to male donor combination					0.54
No	1561 (80.3)	761 (80.7)	599 (79.1)	201 (82)	
Yes	384 (19.7)	182 (19.3)	158 (20.9)	44 (18)	
Unknown	13	6	5	2	
Type of donor					0.36
Haploidentical	250 (12.8)	117 (12.3)	94 (12.3)	39 (15.8)	
Matched related donor	662 (33.8)	335 (35.3)	245 (32.2)	82 (33.2)	
Unrelated donor	1046 (53.4)	497 (52.4)	423 (55.5)	126 (51)	
CMV status/patient					**0.05**
Negative	721 (37.7)	323 (35)	304 (40.7)	94 (38.8)	
Positive	1192 (62.3)	601 (65)	443 (59.3)	148 (61.2)	
Unknown	45	25	15	5	
CMV status/donor					0.31
Negative	939 (48.9)	452 (48.8)	378 (50.3)	109 (44.7)	
Positive	982 (51.1)	474 (51.2)	373 (49.7)	135 (55.3)	
Unknown	37	23	11	3	
CMV status donor to patient					**0.05**
Negative to negative	539 (28.5)	240 (26.4)	226 (30.5)	73 (30.4)	
Negative to posistive	384 (20.3)	204 (22.4)	145 (19.6)	35 (14.6)	
Positive to negative	174 (9.2)	78 (8.6)	76 (10.3)	20 (8.3)	
Positive to positive	792 (41.9)	387 (42.6)	293 (39.6)	112 (46.7)	
Unknown	69	40	22	7	
In vivo T-cell depletion					0.30
No	849 (44)	413 (44.3)	318 (42.4)	118 (48)	
Yes	1080 (56)	520 (55.7)	432 (57.6)	128 (52)	
Unknown	29	16	12	1	
In vivo T-cell depletion					
ATG	833 (43.2)	398 (42.7)	338 (45.1)	97 (39.4)	-
ATG + alemtuzumab	7 (0.4)	5 (0.5)	2 (0.3)	0 (0)	-
Alemtuzumab	240 (12.4)	117 (12.5)	92 (12.3)	31 (12.6)	-
No T-cell depletion	849 (44)	413 (44.3)	318 (42.4)	118 (48)	-
Unknown	29	16	12	1	-
Conditioning regimen					**0.012**
RIC	1160 (60.2)	533 (57.1)	465 (61.9)	162 (66.7)	
MAC	767 (39.8)	400 (42.9)	286 (38.1)	81 (33.3)	
Missing	31	16	11	4	
PTCY					0.239
No	1518 (79.7)	736 (79.9)	598 (80.7)	184 (75.7)	
Yes	387 (20.3)	185 (20.1)	143 (19.3)	59 (24.3)	
Unknown	53	28	21	4	
Ann Arbor stage at diagnosis					** < 0.0001**
I-II	56 (12.4)	30 (15.5)	9 (4.7)	17 (25)	
III	128 (28.3)	44 (22.8)	73 (38)	11 (16.2)	
IV	269 (59.4)	119 (61.7)	110 (57.3)	40 (58.8)	
Unknown	1505	756	570	179	
Performed CT scan at allo-SCT					0.52
No	162 (15.4)	75 (16.4)	57 (13.8)	30 (16.2)	
Yes	892 (84.6)	381 (83.6)	356 (86.2)	155 (83.8)	
Unknown	904	493	349	62	
Performed PET scan at allo-SCT					0.18
Negative	458 (56.3)	192 (53.3)	175 (56.8)	91 (62.3)	
Positive	356 (43.7)	168 (46.7)	133 (43.2)	55 (37.7)	
Missing	1144	589	454	101	
Number of lines prior to allo-SCT					0.92
1	301 (23)	134 (23.1)	116 (22.6)	51 (23.6)	
2	431 (32.9)	196 (33.8)	163 (31.7)	72 (33.3)	
3 or more	578 (44.1)	250 (43.1)	235 (45.7)	93 (43.1)	
Unknown	648	369	248	31	
Disease status at allo-SCT					**0.0117**
CR/PR	1480 (79.8)	707 (78)	568 (79.6)	205 (86.9)	
Progressive or stable disease	374 (20.2)	198 (21.9)	145 (20.3)	31 (13.1)	
Unknown	104	44	49	11	
Remission status at allo-SCT					**0.0061**
CR	967 (52.2)	441 (48.5)	384 (53.9)	142 (60.2)	
PR	513 (27.7)	266 (29.4)	184 (25.8)	63 (26.7)	
Progressive or stable disease	374 (20.2)	198 (21.9)	145 (20.3)	31 (13.1)	
Unknown	104	44	49	11	
International Prognostic Index at first diagnosis					** < 0.0001**
Low risk (0–1)	92 (15.8)	36 (15.5)	20 (8.5)	36 (30.8)	
Low-intermediate risk (2)	153 (26.2)	62 (26.7)	60 (25.5)	31 (26.5)	
High-intermediate risk (3)	208 (35.6)	73 (31.5)	102 (43.4)	33 (28.2)	
High risk (4 or 5)	131 (22.4)	61 (26.3)	53 (22.6)	17 (14.5)	
Unknown	1374	717	527	130	
Karnofsky Index at allo-SCT					0.68
< 90	570 (31)	285 (31.8)	219 (30.7)	66 (28.9)	
> = 90	1267 (69)	610 (68.2)	495 (69.3)	162 (71.1)	
Unknown	121	54	48	19	
Sorror (HCT-CI) index					0.17
0	697 (53.7)	322 (53.1)	275 (55.2)	100 (51.5)	
1 to 2	288 (22.2)	148 (24.4)	93 (18.7)	47 (24.2)	
3 +	313 (24.1)	136 (22.4)	130 (26.1)	47 (24.2)	
Unknown	660	343	264	53	
TBI					0.29
No	1454 (74.4)	690 (73.1)	572 (75.1)	192 (77.7)	
Yes	499 (25.6)	254 (26.9)	190 (24.9)	55 (22.3)	
Unknown	5	5	0	0	
Conditioning					
BEAM	37 (1.9)	13 (1.4)	21 (2.8)	3 (1.2)	-
BuCy/BuCyFlu based	239 (12.3)	117 (12.6)	99 (13)	23 (9.4)	-
BuFlu based	502 (25.9)	224 (24.1)	205 (26.9)	73 (29.8)	-
CyFlu	145 (7.5)	70 (7.5)	52 (6.8)	23 (9.4)	-
FluMel based	341 (17.6)	167 (18)	131 (17.2)	43 (17.6)	-
Other	84 (4.3)	45 (4.8)	29 (3.8)	10 (4.1)	-
TBI based	499 (25.8)	254 (27.3)	190 (25)	55 (22.4)	-
Treo based	89 (4.6)	40 (4.3)	34 (4.5)	15 (6.1)	-
Unknown	22	19	1	2	-
GvHD prophylaxis					-
CSA based	305 (15.8)	145 (15.6)	119 (15.9)	41 (16.7)	-
CSA MMF based	466 (24.2)	194 (20.8)	217 (28.9)	55 (22.4)	-
CSA MTX/ MMF + MTX based	487 (25.3)	258 (27.7)	166 (22.1)	63 (25.6)	-
MMF/ MTX based	202 (10.5)	109 (11.7)	72 (9.6)	21 (8.5)	-
Other	81 (4.2)	41 (4.4)	33 (4.4)	7 (2.8)	-
PTCY based	387 (20.1)	185 (19.8)	143 (19.1)	59 (24)	-
Unknown	30	17	12	1	-
Previous auto-SCT					**0.0453**
No	1102 (56.3)	559 (58.9)	417 (54.7)	126 (51)	
Yes	856 (43.7)	390 (41.1)	345 (45.3)	121 (49)	
* Up-front*	264 (30.8)	107 (61.1)	115 (73.2)	42 (57.5)	-
* Salvage*	141 (16.5)	68 (38.8)	42 (26.8)	31 (42.5)	-
Unknown	451 (52.7)	215 (55.1)	188 (54.5)	48 (25.6)	-

Allo-SCT, allogeneic stem cell transplantation; IQR, interquartile range; ALK-negative ALCL, anaplastic lymphoma kinase-negative anaplastic large cell lymphoma; AITL, angioimmunoblastic T-cell lymphoma; PTCL NOS, peripheral T-cell lymphoma not otherwise specified; ATG, anti-thymocyte globulin; RIC, reduced-intensity conditioning; MAC, myeloablative conditioning; PTCY, post-transplant cyclophosphamide; CT, computed tomography; PET, positron emission tomography; CR, complete remission; PR, partial remission; SD/PD, stable disease/progressive disease; IPI, international prognostic index; GvHD, graft-versus-host disease; CSA, cyclosporine; MMF, mycophenolate mofetil; MTX, methotrexate; auto-SCT, autologous stem cell transplantation; Results expressed as n (%) unless otherwise stated

### Transplantation outcomes

**Fig. 1:**
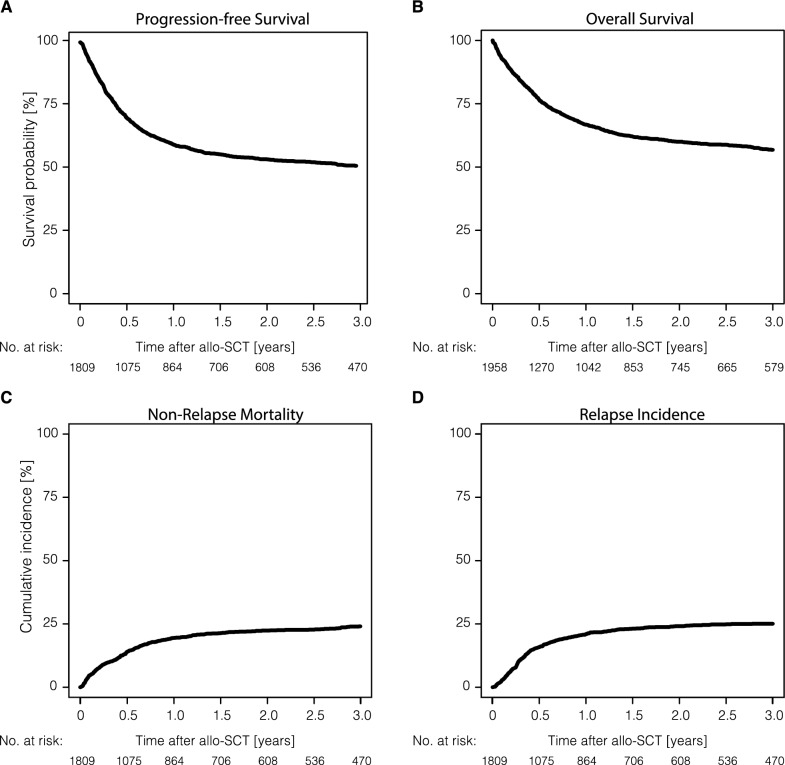
Outcomes of all study patients undergoing allo-SCT. Allo-SCT, allogeneic stem cell transplantation

**Fig. 2:**
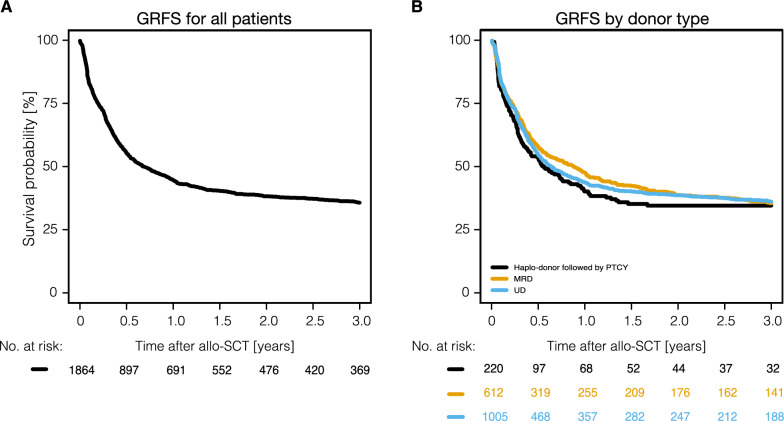
Graft-versus-host disease–free, relapse-free survival (GRFS) of all study patients undergoing allo-SCT and depending on donor type. Allo-SCT, allogeneic stem cell transplantation. Haplo, haploidentical; PTCY, post-transplant cyclophosphamide; MRD, matched-related donor; UD, unrelated donor

Major outcomes of patients receiving allo-SCT are shown in Figs. 1, 2 and Table 2. With a median follow-up of 3.1 years (95% CI: 3.0–3.4 years), RI was 21.0% (95% CI: 19.1–23.0%) and 25.1% (95% CI: 22.9–27.3%) at 1 and 3 years; NRM was 19.5% (95% CI: 17.6–21.4%) and 24.1% (95% CI: 21.9–26.3%) at 1 and 3 years, respectively. The rates of PFS and OS were 59.6% (95% CI: 57.1–61.9%) and 66.7% (95% CI: 64.5–68.9%) at one year, and 50.9% (95% CI: 48.3–53.4%) and 56.8% (95% CI: 54.3–59.2%) at 3 years, respectively. The incidence of aGvHD grades II-IV at day 100 was 31.1% (95% CI: 29.0–33.3%), while the cumulative incidences of cGVHD at 1 year and 3 years posttransplant were reported as 26.3% (95% CI: 24.1–28.5%) and 33.9% (95% CI: 31.4–36.3%), respectively. Extensive cGvHD was reported as 11.5% (95% CI: 10.0–13.2%) and 15.8% (95% CI: 13.9–17.8%) at 1 year and 3 years after allo-SCT. The rates of GRFS were 44.7% (95% CI: 42.3–47.1%) at one year, and 35.8% (95% CI: 33.4–38.2%) at 3 years, respectively. At last follow-up, 780 patients (39.8%) had died with allo-SCT-related death (including GvHD and infectious complications) being the most frequent cause in 374 patients (50.2%), followed by lymphoma in 289 patients (38.8%), secondary malignancy in 66 patients (8.8%), or other causes (n = 16; 2.1%).

**Table 2:** Post-transplantation outcomes for patients with major T-cell lymphomas

	Total	PTCL NOS	AITL	ALK-neg. ALCL	P-value
Outcomes	Estimation (95% CI)	Estimation (95% CI)	Estimation (95% CI)	Estimation (95% CI)	
Median FU, y (range)	3.1 (3—3.4)	3.6 (3—4.1)	3.1 (2.9—3.6)	2.1 (1.8—2.9)	
OS (1 y)	66.7 (64.5—68.9)	64.2 (60.9—67.3)	68.8 (65.2—72.1)	70 (63.4—75.7)	**0.02**
OS (2 y)	60 (57.6—62.3)	55.5 (52—58.9)	63.9 (60.1—67.4)	65.8 (58.9—71.9)	
OS (3 y)	56.8 (54.3—59.2)	52.9 (49.3—56.3)	60.7 (56.7—64.4)	59.7 (52—66.6)	
PFS (1 y)	59.6 (57.1—61.9)	56.6 (53—60)	63.1 (59.2—66.7)	59.7 (52.7—66.1)	**0.004**
PFS (2 y)	53.5 (51—56)	48.8 (45.1—52.4)	59.4 (55.4—63.2)	53.2 (45.9—60)	
PFS (3 y)	50.9 (48.3—53.4)	46.1 (42.4—49.7)	57.2 (53.1—61.1)	49.5 (41.8—56.7)	
RI (1 y)	21 (19.1—23)	24.1 (21.2—27.1)	15.7 (13—18.6)	25.4 (19.7—31.5)	** < 0.0001**
RI (2 y)	24.1 (22—26.3)	28.5 (25.3—31.7)	16.9 (14.1—19.9)	30 (23.7—36.5)	
RI (3 y)	25.1 (22.9—27.3)	29.8 (26.5—33.1)	17.4 (14.5—20.5)	30.9 (24.4—37.6)	
NRM (1 y)	19.5 (17.6—21.4)	19.3 (16.6—22.1)	21.2 (18.1—24.4)	14.9 (10.4—20.1)	0.08
NRM (2 y)	22.4 (20.3—24.5)	22.7 (19.8—25.8)	23.7 (20.4—27.1)	16.8 (11.9—22.3)	
NRM (3 y)	24.1 (21.9—26.3)	24.1 (21.1—27.3)	25.5 (22—29)	19.6 (14—25.9)	
aGvHD-II/IV (100 d)	31.1 (29—33.3)	28.3 (25.3—31.4)	32.5 (29—36.1)	37.4 (31.1—43.7)	**0.02**
aGvHD-III/IV (100 d)	11.4 (9.9—13)	9.9 (8—12.1)	12.3 (10—14.9)	14 (9.8—18.9)	0.12
cGvHD (1 y)	26.3 (24.1—28.5)	21.4 (18.5—24.4)	31.3 (27.6—35)	29.5 (23.2—36.2)	**0.0001**
cGvHD (2 y)	32 (29.7—34.4)	27.3 (24.1—30.6)	37.4 (33.4—41.3)	33.6 (26.8—40.5)	
cGvHD (3 y)	33.9 (31.4—36.3)	28.9 (25.6—32.3)	39.8 (35.7—43.8)	34.5 (27.6—41.5)	
cGvHD ext (1 y)	11.5 (10—13.2)	9.6 (7.6—11.9)	13.2 (10.6—16.1)	13.7 (9.2—19)	0.08
cGvHD ext (2 y)	14.6 (12.8—16.5)	12.4 (10.1—15)	16.9 (13.9—20.1)	15.7 (10.8—21.4)	
cGvHD ext (3 y)	15.8 (13.9—17.8)	13.6 (11.1—16.3)	17.9 (14.8—21.3)	17.6 (12.2—23.8)	
GRFS (1 y)	44.7 (42.3—47.1)	43.8 (40.4 – 47.2)	46.4 (42.5—50.1)	43.2 (36.5—49.8)	0.08
GRFS (2 y)	38.3 (35.9 – 40.7)	36.4 (33.0—39.7)	41.1 (37.2—44.9)	37.4 (30.6 – 44.1)	
GRFS (3 y)	35.8 (33.4—38.2)	33.4 (30.1—36.8)	39.0 (35.1—42.8)	35.6 (28.8—42.5)	
Death at last follow-up, n (%)	780 (39.8)	413 (43.5)	283 (37.1)	84 (24.2)	-
Cause of death					
allo-SCT-related	374 (50.2)	185 (46.8)	157 (58.4)	32 (39.5)	-
Lymphoma-related	289 (38.8)	176 (44.6)	73 (27.1)	40 (49.4)	
Secondary malignancy	66 (8.8)	28 (7.1)	29 (10.8)	9 (11.1)	
Other	16 (2.1)	6 (1.5)	10 (3.7)	0 (0)	
Unknown	35	18	14	3	

Allo-SCT, allogeneic stem cell transplantation; ALK-negative ALCL, anaplastic lymphoma kinase-negative anaplastic large cell lymphoma; AITL, angioimmunoblastic T-cell lymphoma; PTCL NOS, peripheral T-cell lymphoma not otherwise specified; FU, follow-up; y, year(s); d, days; OS, overall survival; PFS, progression-free survival; RI, relapse incidence; NRM, non-relapse mortality; aGvHD, acute graft-versus-host disease; cGvHD, chronic graft-versus-host disease; ext, extensive

### *Univariate analyses *in* patients with allo-SCT*

**Fig. 3:**
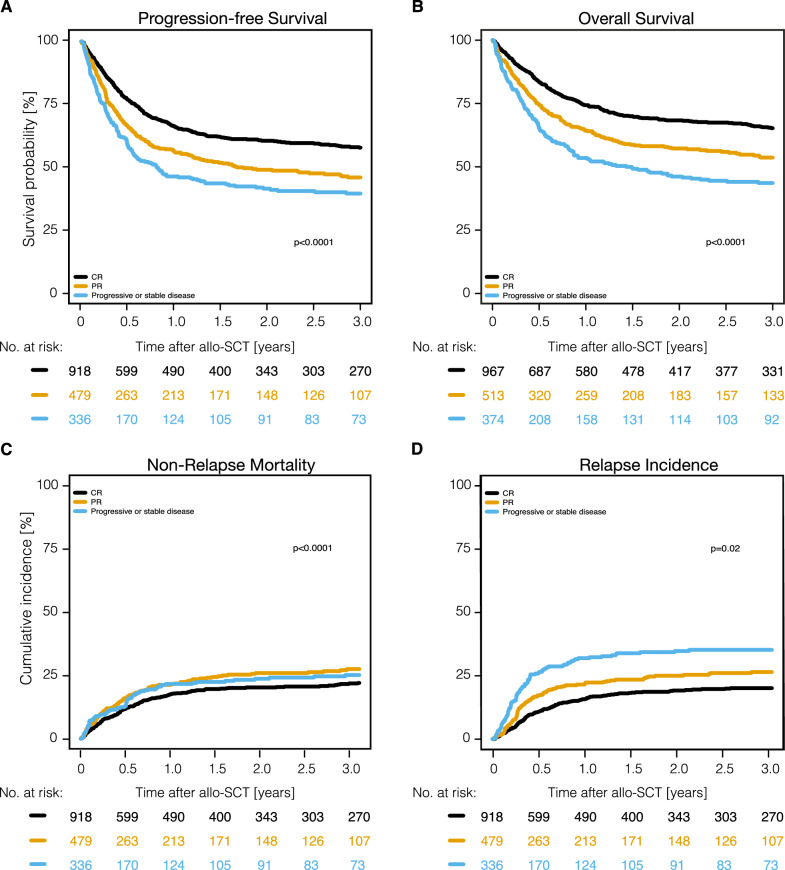
Outcomes of allo-SCT according to remission status at SCT (CR vs. PR vs. SD/PD). Allo-SCT, allogeneic stem cell transplantation; CR, complete remission; PR, partial remission; SD/PD, stable disease/progressive disease

OS, PFS, RI, and NRM across subgroups are shown in Tables 2, 3, Figs. 2, 3 and Supplemental Fig. S1-6. Patients aged ≤ 53 years survived better than those aged > 53 years due to a lower 3-year NRM [18.6% (95% CI: 15.8–21.5%) vs. 28.9% (95% CI: 25.7–32.0%) (p < 0.0001)] while the rate of RI was similar (Table 3). Patients undergoing allo-SCT in CR demonstrated significantly better PFS and OS than PR and SD/PD patients following significantly lower 3-year RI: 20.2% (95% CI: 17.4–23.1%) vs. 26.5% (95% CI: 22.3–30.9%) vs. 35.3% (95% CI: 29.9–40.7%) (p < 0.0001) (Table 3, Fig. 2). Notably, no significant differences in survival were documented between CR and CMR, as well as PR and non-CMR patients (Supplemental Fig. S1-2). Patients undergoing allo-SCT after only 1L of treatment demonstrated significantly better OS than those receiving allo-SCT in 2L or later (3L +): 68.1% (95% CI: 61.7–73.7%) vs. 56.7% (95% CI: 51.2–61.9%) vs. 55.0% (95% CI: 50.3–59.5%) (p < 0.0001). This was partly due to a significantly lower 3-year NRM in comparison to 2L and 3L + groups [14.6% (95% CI: 10.4–19.4%) vs. 26.7% (95% CI: 21.9–31.7%) vs. 25.3% (95% CI: 21.3–29.5%) (p = 0.0007)] while 3-year PFS and RI did not differ significantly among these patients (p ≥ 0.09 for both) (Table 3 and Supplemental Fig. S3). Auto-SCT preceding allo-SCT in 43.7% of patients did not have a significant impact on any outcomes after allo-SCT including NRM (Table 3, Supplemental Fig. S4).

**Table 3:** Univariate analysis of factors influencing OS, PFS, RI, and NRM post-allo-SCT

Outcome	3-year probability [95% CI]			P-value
	**Sex**			
	**Male**	**Female**	**-**	
OS	56.3% [53.3–59.3]	57.8% [53.5–61.9]	-	0.43
PFS	50.3% [47.1–53.4]	52% [47.5–56.4]	-	0.335
RI	26.3% [23.6–29]	22.7% [19.2–26.4]	-	0.11
NRM	23.4% [20.8–26.1]	25.2% [21.5–29.1]	-	0.588
	**Age at allo-SCT (years)**			
	**18 to 53**	**> 53**	**-**	
OS	61.4% [57.8–64.9]	52.7% [49.2–56.1]	-	** < 0.0001**
PFS	53.4% [49.5–57]	48.7% [45.1–52.2]	-	**0.023**
RI	28.1% [24.8–31.4]	22.4% [19.7–25.3]	-	**0.018**
NRM	18.6% [15.8–21.5]	28.9% [25.7–32]	-	** < 0.0001**
	**IPI score at diagnosis**			
	**Low-intermediate (0–2 points)**	**Intermediate-high (3–5 points)**	**-**	
OS	63.1% [55.7–69.7]	59.9% [53.7–65.6]	-	0.464
PFS	55.7% [47.9–62.8]	52.4% [46.1–58.4]	-	0.553
RI	25.9% [19.6–32.6]	24% [19.1–29.3]	-	0.769
NRM	18.4% [13.1–24.4]	23.6% [18.6–28.9]	-	0.249
	**Delay diagnosis to allo-SCT in months**			
	** ≤ 12 months**	** > 12 months**	-	
OS	55.4% [51.3–59.3]	57.6% [54.4–60.6]	-	0.0511
PFS	50.4% [46.2–54.5]	51.1% [47.8–54.3]	-	0.206
RI	25.7% [22.2–29.3]	24.8% [22.1–27.5]	-	0.424
NRM	23.9% [20.5–27.4]	24.1% [21.4–26.9]	-	0.669
	**Prior auto-SCT**			
	**Yes**	**No**	-	
OS	58.1% [54.3–61.7]	55.7% [52.4–58.9]	-	0.121
PFS	50.9% [46.9–54.6]	50.9% [47.4–54.3]	-	0.788
RI	27.1% [23.8–30.5]	23.5% [20.7–26.4]	-	0.116
NRM	22% [19–25.3]	25.6% [22.7–28.6]	-	0.062
	**Disease status at allo-SCT**			
	**CR**	**PR**	**SD/PD**	
OS	65.2% [61.7–68.5]	53.7% [48.6–58.4]	43.6% [38–49.1]	** < 0.0001**
PFS	57.8% [54.2–61.3]	46% [40.9–51]	39.6% [33.9–45.3]	** < 0.0001**
RI	20.2% [17.4–23.1]	26.5% [22.3–30.9]	35.3% [29.9–40.7]	** < 0.0001**
NRM	22% [19.1–25]	27.5% [23.2–32]	25.1% [20.3–30.3]	**0.0214**
	**Number of lines prior to allo-SCT**			
	**1**	**2**	**3 or more**	
OS	68.1% [61.7–73.7]	56.7% [51.2–61.9]	55% [50.3–59.5]	**0.0002**
PFS	57.3% [50.6–63.4]	50.8% [45.1–56.3]	48.6% [43.7–53.4]	0.0898
RI	28.2% [22.6–34.1]	22.5% [18.2–27.1]	26.1% [22–30.2]	0.359
NRM	14.6% [10.4–19.4]	26.7% [21.9–31.7]	25.3% [21.3–29.5]	0.0007
	**Myeloablative regimen**			
	**No**	**Yes**	**-**	
OS	58.3% [55.1–61.4]	54.7% [50.6–58.6]	-	0.0769
PFS	53.4% [50.1–56.6]	46.9% [42.7–51]	-	**0.0147**
RI	23% [20.4–25.8]	27.8% [24.2–31.5]	-	0.0626
NRM	23.6% [20.9–26.4]	25.3% [21.8–28.9]	-	0.336
	**Use of TBI**			
	**No**	**Yes**	**-**	
OS	56.7% [53.8–59.5]	56.9% [51.9–61.6]	-	0.683
PFS	50.6% [47.6–53.5]	52% [46.9–56.8]	-	0.878
RI	25.3% [22.8–27.9]	23.8% [19.8–28]	-	0.659
NRM	24.1% [21.6–26.6]	24.2% [20.1–28.6]	-	0.784
	**Type of donor**			
	**Haploidentical**	**MRD**	**UD**	
OS	55.8% [48.8–62.3]	59.8% [55.5–63.9]	55.2% [51.7–58.5]	**0.012**
PFS	52.5% [45.2–59.3]	52.7% [48.3–56.9]	49.4% [45.8–52.9]	0.439
RI	18.7% [13.5–24.6]	28.9% [25.1–32.8]	24.1% [21.2–27]	**0.009**
NRM	28.8% [22.7–35.2]	18.3% [15.1–21.8]	26.5% [23.5–29.7]	** < 0.0001**

Allo-SCT, allogeneic stem cell transplantation; OS, overall survival; PFS, progression-free survival; RI, relapse incidence; NRM, non-relapse mortality; IPI, international prognostic index; CR, complete remission; TBI, total body irradiation; MRD, matched related donor; UD, unrelated donor

### *Different *outcomes* in major entities*

**Fig. 4:**
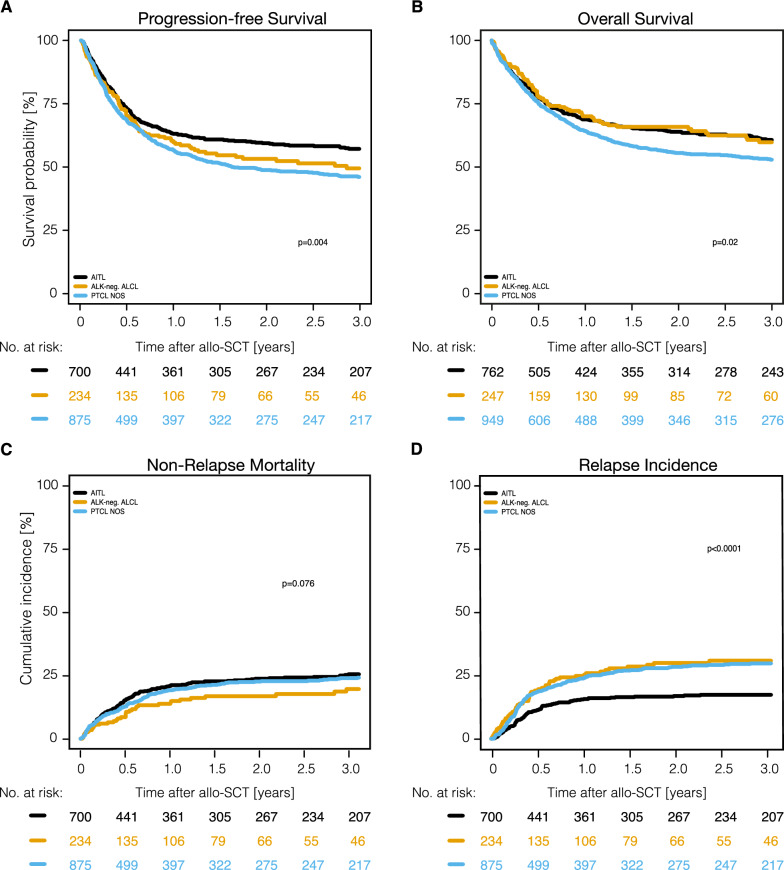
Outcomes of allo-SCT depending on histology. Allo-SCT, allogeneic stem cell transplantation; ALK-negative ALCL, anaplastic lymphoma kinase-negative anaplastic large cell lymphoma; PTCL NOS, peripheral T-cell lymphoma not otherwise specified; AITL, angioimmunoblastic T-cell lymphoma

OS, PFS, RI, and NRM for the major entities are shown in Fig. 4, Supplemental Fig. S5-6 and Table 2. While the 3-year NRM rate did not differ significantly across all three entities (24.1%, range, 21.9–26.3%) (p = 0.08), 3-year RI was significantly lower in AITL [(RI: 17.4% (95% CI: 14.5–20.5%)] in comparison to PTCL NOS [(RI: 29.8% (95% CI: 26.5–33.1%)] and ALK-negative ALCL [(RI: 30.9% (95% CI: 24.4–37.6%)] (p < 0.0001). Entity-specific analyses showed the highest 3-year PFS-rates in patients with AITL [(PFS 57.2% (95% CI, 53.1%–61.1%). 3-year PFS rates were 46.1% (95% CI, 42.4%–49.7%), and 49.5% (95% CI, 45.8%–54.5%) in PTCL NOS and ALK-negative ALCL, respectively (p = 0.004). The highest 3-year OS rates were observed in AITL and ALK-negative ALCL [(60.7% (95% CI: 56.7–64.4%), and 59.7% (95% CI: 52.0–66.6%)], and the lowest in PTCL NOS [(52.9% (95% CI: 49.3–56.3%)] (p < 0.0001) (Table 2, Fig. 4). 3-year GRFS was similar among all three entities: 39% (95% CI: 35.1–42.2%), 35.6% (95% CI: 28.8–42.5%), and 33.4% (95% CI: 30.1–36.8%) for patients with AITL, ALK-neg. ALCL and PTCL NOS, accordingly (p > 0.05) (Table 2**)**. Notably, 3-year GRFS was almost identical in patients allografted from haploidentical donors [34.6% (95% CI: 27.8–41.4%)], matched related [35.5% (95% CI: 31.5–39.6%)] and unrelated donors [36.2% (95% CI: 32.9–39.5%)] (Fig. 2).

Patients with ALK-negative ALCL or AITL showed better survival and lower RI compared to patients with PTCL NOS when undergoing allo-SCT in CR/CMR (Supplemental Fig. S5). As expected, outcomes were worse in patients allografted with PR or non-CMR (Supplemental Fig. S6). Major outcomes were numerically better for patients undergoing allo-SCT with SD than with PD: 3-year PFS was 50.2% (95% CI: 31.7–66.1%) vs. 38.2% (95% CI: 32.1–44%) (p = 0.16) and 3-year OS 52.2% (95% CI: 35.1–66.7%) vs. 42.4% (95% CI: 36.5–48.3%) (p = 0.2). Three-year RI was 27.3% (95% CI: 12.9–44%) vs. 36.4% (95% CI: 30.6–42.2%) (p = 0.14), 3-year NRM 22.5% (95% CI: 10.3–37.7%) vs. 36.4% (95% CI: 30.6–42.2%) (p = 0.996) (Supplemental Fig. S7). Notably, patients with AITL showed a significantly lower RI [26.1% (95% CI: 18.1–34.8)] compared to patients with PTCL NOS [39.1% (95% CI: 31.6–46.5)] or ALK-neg. ALCL [52.8% (95% CI: 31.6–70.1] when undergoing allo-SCT in SD/PD (p = 0.002) (Supplemental Fig. S8).

### *Prognostic *factors* in patients undergoing allo-SCT*

**Fig. 5:**
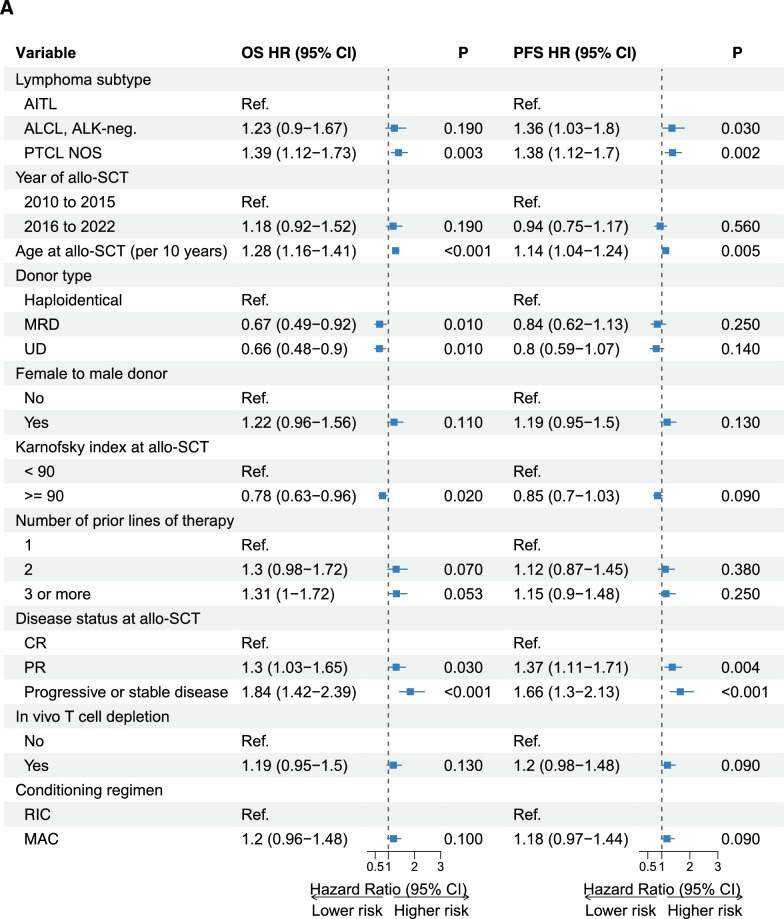

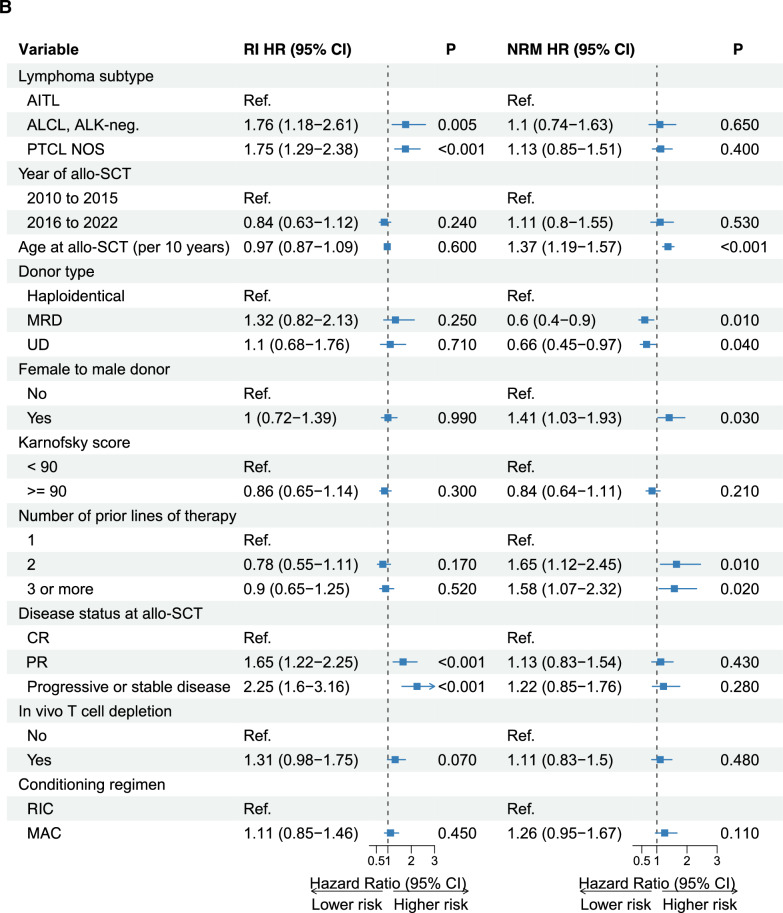
Prognostic factors influencing the outcomes of allo-SCT (**A**: PFS/OS; **B**: RI/NRM) in a multivariate cox-regression model. Allo-SCT, allogeneic stem cell transplantation. AITL, angioimmunoblastic T-cell lymphoma; ALK-negative ALCL, anaplastic lymphoma kinase-negative anaplastic large cell lymphoma; PTCL NOS, peripheral T-cell lymphoma not otherwise specified; CR, complete remission; PR, partial remission; SD/PD, stable disease/progressive disease; BEAM, carmustine, etoposide, cytarabine, melphalan

To investigate factors influencing major outcomes of allo-SCT, a multivariate model was used which included the following variables: lymphoma histology, time period of SCT (2010–2015 vs. 2016–2022), patient age, type of donor, KPS, number of prior therapy lines at SCT, remission status at SCT, in vivo T-cell depletion, and conditioning (Fig. 5A, B; Supplemental Tables S5A-B).

### Progression Free Survival

Increased age (HR 1.14, 95% CI 1.04–1.24, p = 0.005), and no CR at allo-SCT (HR 1.37, 95% CI 1.11–1.71, p = 0.004 for PR; HR 1.66, 95% CI 1.3–2.13, p < 0.001 for SD/PD) were associated with lower PFS. Patients with PTCL NOS (HR 1.38, 95% CI 1.12–1.7, p = 0.002) and ALK-negative ALCL (HR 1.36, 95% CI 1.03–1.8, p = 0.03) showed a lower PFS when compared to patients with AITL (Fig. 5A).

### Overall survival

On multivariate analysis, KPS ≥ 90% (HR 0.78, 95% CI 0.63–0.96, p = 0.02), having a matched related (HR 0.67, 95% CI 0.49–0.92, p = 0.01) or matched unrelated donor (HR 0.66, 95% CI 0.48–0.9, p = 0.01) were associated with significantly better OS. Increased age (HR 1.28, 95% CI 1.16–1.41, p < 0.001), PR at allo-SCT (HR 1.30, 95% CI 1.03–1.65, p = 0.030), SD/PD at allo-SCT (HR 1.84, 95% CI 1.42–2.39, p < 0.001) were associated with lower OS. When compared with AITL as the reference group, patients with PTCL NOS (HR 1.43, 95% CI 1.16–1.177, p < 0.001) showed a lower OS while the difference to ALK-negative ALCL was not statistically significant (HR 1.24, 95% CI 0.91–1.67, p = 0.17) (Fig. 5A).

### *Relapse *incidence

Multivariate analysis showed that no CR at allo-SCT (PR: 1.65, 95% CI 1.22–2.25, p < 0.001; SD/PD: HR 2.25, 95% CI 1.6–3.16, p < 0.001) and histology other than AITL (HR 1.76, 95% CI 1.18–2.61, p = 0.005 for ALK-negative ALCL; HR 1.75, 95% CI 1.29–2.38, p < 0.001 for PTCL NOS) were associated with an increased risk of RI (Fig. 5B).

### *Non-*relapse* mortality*

Age (by ten year increments) at allo-SCT (HR 1.37, 95% CI 1.19–1.57, p < 0.001), female to male donor (HR 1.41, 95% CI 1.03–1.93, p = 0.03), and having a haploidentical donor was associated with an increased risk of NRM (matched related donor: HR 0.60, 95% CI 0.40–0.90, p = 0.01; unrelated donor: HR 0.66, 95% CI 0.45–0.97, p = 0.04). Patients allografted in 2L (HR 1.65, 95% CI 1.12–2.45, p = 0.01) or with 3 or more previous treatment lines (HR 1.58, 95% CI 1.07–2.32, p = 0.02) showed a higher risk of NRM compared to patients allografted after 1L (Fig. 5B).

## Discussion

This study reports outcomes of large numbers of patients with any of the major T-cell lymphoma entities (PTCL NOS, AILT, ALK-negative ALCL) and allografted in recent years. Overall, allo-SCT resulted in 3-year GRFS-, PFS-, and OS-rates of 39%, 50.9% and 56.8%; 3-year RI and NRM was 25.1% and 24.1%, respectively. These data match very well the long-term outcomes reported for the AATT study, a randomized phase III study, comparing up-front auto- and allo-SCT in patients with T-cell lymphoma [12]. Because of the large patient numbers investigated, in this study we were able to separately analyse survival, RI, and NRM in patients with different histologies, having failed different lines of therapy, having different donors, presenting with differing disease status prior to transplantation, receiving various conditioning, and identify prognostic factors important for patients undergoing allo-SCT.

Surprisingly, the survival after allo-SCT differed significantly between major entities with the lowest risk of relapse and best progression-free survival seen in AITL patients. This finding is in line with reports investigating targeted therapies as first- and second-line therapies [16, 17], analyses of auto-SCT [18] and three larger retrospective analyses after allo-SCT [19–21]. Importantly, we show that the better survival of AITL patients is driven by a significantly lower RI, also when comparing CR/CMR, PR and SD/PD patients separately.

The number of patients receiving allo-SCT after one line of therapy only increased over recent years and showed the most favorable survival mostly because of the significantly lower NRM compared to patients allografted in second or later line of therapy. The French transplantation society (SFGM-TC) also reported lower NRM in patients undergoing allo-SCT up-front as compared to second-line allo-SCT (24% vs. 30%) [22]. Nonetheless, following the results of the AATT study and recent international guidelines, allo-SCT is generally not recommended for consolidation of remission after one line of therapy only [3, 10, 12, 23, 24]. Autologous transplantation remains the preferred option in such cases [18]. In the large retrospective analysis by Hamadani et al., survival after allo-SCT significantly decreased in patients with ≥ 3 lines of therapy, but not when one and two prior lines of treatment were compared [20]. The improved OS after early allo-SCT seen in this cohort may reflect changing transplant modalities but selection and immortal-time bias represent other possibilities to explain the difference.

Relapse rates in all major entities were generally low confirming the existence of a particularly strong GvL effect in T-cell lymphomas [11, 12, 25].

In multivariate analysis, higher age, being not in CR, and having a haploidentical donor were associated with worse outcomes. Higher age has been repeatedly identified as risk factor for poor survival in many reports on allo-SCT not only in lymphoma [19, 20]. As expected, CR at transplant was the most favorable remission status prior to transplantation. However, patients in PR still experienced a 3-year PFS and OS of 46% and 53.7%, respectively, suggesting that allo-SCT can also provide durable remissions among these patients. Outcomes were less encouraging in patients who were not in remission before allo-SCT; however, more than one-third of such patients still can expect to survive long-term (3-year PFS 39.6% and OS 43.6%). This is in line with published data from registry and single center analyses as well as long-term follow up of AATT patients [12, 20, 26–28]. Nonetheless, transplant candidates before undergoing allo-SCT in SD/PD should be evaluated carefully. Younger patients presenting with a good performance status and limited activity of the lymphoma (as evidenced e.g. by moderate LDH levels and total metabolic volume), even if documented as SD or PD, should not be refused to go to allo-SCT, in particular, as promising alternative modalities for such patients are missing.

We compared the disease status reported by CT- and PET-CT scan before allo-SCT without finding significant differences in outcomes, regardless of whether CT or PET-CT had been used for imaging prior to transplantation. Similar observations were reported for patients undergoing auto-SCT [18]. This finding is important because the comparison of transplant outcomes documented by different imaging techniques has repeatedly been questioned. If identification of patients refractory to conventional first-line therapy by early interim PET scan (iPET2 or 3) [29, 30] will help bringing more patients to allo-SCT with more favorable results is addressed by a Chinese study the final results of which are pending (NCT06509945).

As mentioned above, the GvL-effect exerted by donor T-cells seems particularly strong in T-cell lymphoma and it may be less important than in other entities to destroy as many lymphoma cells as possible by conditioning. While others reported that in patients with PTCL, MAC is associated with worse outcomes than RIC, mainly due to increased NRM [11, 22, 31, 32], we could not confirm this finding. In this study, NRM was generally lower (around 20% at one year) than reported for recent prospective studies [3, 25] which used only MAC, but we were unable to confirm favorable outcomes after RIC as compared to MAC. Among others, the good performance status of our patients, their relatively young age, and the fact that many patients were not in CR at allo-SCT may have contributed to this finding. In the prospective AATT study, NRM was 31% following myeloablative conditioning even in the up-front setting [12]. In another prospective study, investigating another myeloablative conditioning regimen in patients with aggressive lymphoma NRM was as high as 35% [25]. Thus, NRM after MAC is unacceptably high and we recommend to use RIC in all patients with the possible exception of those transplanted with SD/PD [23].

Over recent years, the number of haploidentical transplants has significantly increased in most entities including lymphoma. In this analysis, haplo-identical transplantation was an independent unfavorable prognostic factor for survival post-allo-SCT. This finding contradicts data published by Hamadani et al. demonstrating comparable outcomes after PTCY-based haplo-SCT and matched donor transplants across major T-cell lymphoma entities [20]. The fact that details of conditioning and GvHD prophylaxis were not fully available in this analysis may partly explain the discrepant findings. Other factors such as selection bias, evolving haploidentical platforms, and differences in regional practice might have contributed to this discrepancy. Of note, 3-year GFRS was similar between haploidentical and matched related/unrelated donor transplants also in our analysis. In practical terms, for patients with T-cell lymphoma, a haplo-identical donor should be accepted whenever a matched related donor and/or matched unrelated donor is not available [23]. This holds true particularly because promising alternatives to allo-SCT are largely missing.

The present study has limitations inherent to any retrospective analysis. Most importantly, we cannot know how many patients initially deemed transplant candidates could not proceed to transplantation because of disease progression or toxicity of induction and salvage therapies. Other limitations are the lack of a centralized pathological review, and the categorization of disease status and treatment response by local investigators. Outcomes may also vary country by country because of differences in pretreatment and supportive care of transplant recipients. These aspects may have implications for generalizability of the results reported here. Data further characterizing our patients allografted with SD/PD such as patterns of progression, details of salvage therapies, and tumor burden present before allo-SCT were not available for this analysis. However, with the large numbers of patients investigated, these shortcomings had to be tolerated and should not change the major conclusions.

## Conclusions

In summary, this analysis of a large international cohort of patients diagnosed with AITL, PTCL NOS, and ALK-negative ALCL demonstrates that allo-SCT provides an unsurpassed high rate of sustainable remissions for such patients. Best results can be expected for patients allografted in chemosensitive disease from a matched related or unrelated donor. However, one-third of patients transplanted in PR or with refractory disease, still can achieve long-term survival after allo-SCT. Therefore, such patients as patients with haplo-identical donors should not be excluded from allo-SCT as long as viable alternatives to allo-SCT are virtually absent. Consistently, patients with AITL showed the most favorable outcomes. The development of targeted agents and immunotherapeutic strategies including chimeric antigen receptor T-cells deserves further attention and may challenge transplantation in the future.

## Supplementary Information

Additional file 1.

Additional file 2.

Additional file 3.

## Data Availability

All data generated or analysed during this study are included in this published article [and its supplementary information files].

## References

[1] Vose J, Armitage J, Weisenburger D. International peripheral T-cell and natural killer/T-cell lymphoma study: pathology findings and clinical outcomes. J Clin Oncol. 2008;26(25):4124–30. 18626005 10.1200/JCO.2008.16.4558

[2] Sibon D, Nguyen DP, Schmitz N, Suzuki R, Feldman AL, Gressin R, et al. ALK-positive anaplastic large-cell lymphoma in adults: an individual patient data pooled analysis of 263 patients. Haematologica. 2019;104(12):e562–5. 31004022 10.3324/haematol.2018.213512 PMC6959172

[3] Schmitz N, Truemper L, Bouabdallah K, Ziepert M, Leclerc M, Cartron G, et al. A randomized phase 3 trial of autologous vs allogeneic transplantation as part of first-line therapy in poor-risk peripheral T-NHL. Blood. 2021;137(19):2646–56. 33512419 10.1182/blood.2020008825 PMC9635528

[4] d’Amore F, Relander T, Lauritzsen GF, Jantunen E, Hagberg H, Anderson H, et al. Up-front autologous stem-cell transplantation in peripheral T-cell lymphoma: NLG-T-01. J Clin Oncol. 2012;30(25):3093–9. 22851556 10.1200/JCO.2011.40.2719

[5] Wulf GG, Altmann B, Ziepert M, D’Amore F, Held G, Greil R, et al. Alemtuzumab plus CHOP versus CHOP in elderly patients with peripheral T-cell lymphoma: the DSHNHL2006-1B/ACT-2 trial. Leukemia. 2021;35(1):143–55. 32382083 10.1038/s41375-020-0838-5

[6] Biasoli I, Cesaretti M, Bellei M, Maiorana A, Bonacorsi G, Quaresima M, et al. Dismal outcome of t-cell lymphoma patients failing first-line treatment: results of a population-based study from the Modena Cancer Registry. Hematol Oncol. 2015;33(3):147–51. 24777784 10.1002/hon.2144

[7] Monica B, Francine MF, Andrei RS, Steven MH, Luigi M, Won Seog K, et al. The outcome of peripheral T-cell lymphoma patients failing first-line therapy: a report from the prospective. Int T-Cell Project Haematol. 2018;103(7):1191–7. 10.3324/haematol.2017.186577 PMC6029527 29599200

[8] Chihara D, Fanale MA, Miranda RN, Noorani M, Westin JR, Nastoupil LJ, et al. The survival outcome of patients with relapsed/refractory peripheral T-cell lymphoma-not otherwise specified and angioimmunoblastic T-cell lymphoma. Br J Haematol. 2017;176(5):750–8. 27983760 10.1111/bjh.14477 PMC5836501

[9] Horwitz S, O’Connor OA, Pro B, Illidge T, Fanale M, Advani R, et al. Brentuximab vedotin with chemotherapy for CD30-positive peripheral T-cell lymphoma (ECHELON-2): a global, double-blind, randomised, phase 3 trial. Lancet. 2019;393(10168):229–40. 30522922 10.1016/S0140-6736(18)32984-2 PMC6436818

[10] NCCN Guidelines. T-Cell Lymphomas. Version 02.2025. https://www.nccn.org/professionals/physician_gls/pdf/t-cell.pdf. Nov 11 2025.

[11] Shumilov E, Levien L, Mazzeo P, Jung W, Leha A, Koch R, et al. Allogeneic stem cell transplantation against aggressive lymphomas: graft-versus-lymphoma effects in peripheral T-cell lymphoma and diffuse large B-cell lymphoma after myeloablative conditioning. Leuk Lymphoma. 2025;66(4):668–79. 39660415 10.1080/10428194.2024.2438805

[12] Tournilhac O, Altmann B, Friedrichs B, Bouabdallah K, Leclerc M, Cartron G, et al. Long-term follow-up of the prospective randomized AATT study (autologous or allogeneic transplantation in patients with peripheral T-cell lymphoma). J Clin Oncol. 2024;42(32):3788–94. 39270145 10.1200/JCO.24.00554

[13] Bacigalupo A, Ballen K, Rizzo D, Giralt S, Lazarus H, Ho V, et al. Defining the intensity of conditioning regimens: working definitions. Biol Blood Marrow Transplant. 2009;15(12):1628–33. 19896087 10.1016/j.bbmt.2009.07.004 PMC2861656

[14] Glucksberg H, Storb R, Fefer A, Buckner CD, Neiman PE, Clift RA, et al. Clinical manifestations of graft-versus-host disease in human recipients of marrow from HL-A-matched sibling donors. Transplantation. 1974;18(4):295–304. 4153799 10.1097/00007890-197410000-00001

[15] Terwey TH, Vega-Ruiz A, Hemmati PG, Martus P, Dietz E, le Coutre P, et al. NIH-defined graft-versus-host disease after reduced intensity or myeloablative conditioning in patients with acute myeloid leukemia. Leukemia. 2012;26(3):536–42. 21869841 10.1038/leu.2011.230

[16] Dupuis J, Bachy E, Morschhauser F, Cartron G, Fukuhara N, Daguindau N, et al. Oral azacitidine compared with standard therapy in patients with relapsed or refractory follicular helper T-cell lymphoma (ORACLE): an open-label randomised, phase 3 study. Lancet Haematol. 2024;11(6):e406–14. 38796193 10.1016/S2352-3026(24)00102-9

[17] Camus V, Thieblemont C, Gaulard P, Cheminant M, Casasnovas R-O, Ysebaert L, et al. Romidepsin plus cyclophosphamide, doxorubicin, vincristine, and prednisone versus cyclophosphamide, doxorubicin, vincristine, and prednisone in patients with previously untreated peripheral T-cell lymphoma: final analysis of the Ro-CHOP trial. J Clin Oncol. 2024;42(14):1612–8. 38364196 10.1200/JCO.23.01687

[18] Shumilov E, Ngoya M, Berning B, Khvedelidze I, Serroukh Y, Wondergem M, et al. Autologous stem cell transplantation in major T-cell lymphoma entities: an analysis by the EBMT Lymphoma Working Party. Hemasphere.in Press; 2025. 10.1002/hem3.70313 PMC12930294 41743267

[19] Kameda K, Kako S, Kim S-W, Usui Y, Kato K, Fukuda T, et al. Autologous or allogeneic hematopoietic cell transplantation for relapsed or refractory PTCL-NOS or AITL. Leukemia. 2022;36(5):1361–70. 35347237 10.1038/s41375-022-01545-w

[20] Hamadani M, Ngoya M, Sureda A, Bashir Q, Litovich CA, Finel H, et al. Outcome of allogeneic transplantation for mature T-cell lymphomas: impact of donor source and disease characteristics. Blood Adv. 2022;6(3):920–30. 34861680 10.1182/bloodadvances.2021005899 PMC8945300

[21] Epperla N, Ahn KW, Litovich C, Ahmed S, Battiwalla M, Cohen JB, et al. Allogeneic hematopoietic cell transplantation provides effective salvage despite refractory disease or failed prior autologous transplant in angioimmunoblastic T-cell lymphoma: a CIBMTR analysis. J Hematol Oncol. 2019;12(1):6. 30630534 10.1186/s13045-018-0696-z PMC6329157

[22] Mamez AC, Dupont A, Blaise D, Chevallier P, Forcade E, Ceballos P, et al. Allogeneic stem cell transplantation for peripheral T cell lymphomas: a retrospective study in 285 patients from the Société Francophone de Greffe de Moelle et de Thérapie Cellulaire (SFGM-TC). J Hematol Oncol. 2020;13(1):56. 32429979 10.1186/s13045-020-00892-4 PMC7236365

[23] Damaj G, Bazarbachi A, Berning P, Cottereau A-S, Fox CP, Kyriakou C, et al. Allogeneic haematopoietic cell transplantation in peripheral T-cell lymphoma: recommendations from the EBMT Practice Harmonisation and Guidelines Committee. Lancet Haematol. 2025;12(7):e542–54. 40610175 10.1016/S2352-3026(25)00073-0

[24] d’Amore F, Federico M, de Leval L, Ellin F, Hermine O, Kim WS, et al. Peripheral T- and natural killer-cell lymphomas: ESMO-EHA clinical practice guideline for diagnosis, treatment and follow-up. Ann Oncol. 2025;36(6):626–44. 40345949 10.1016/j.annonc.2025.01.023

[25] Glass B, Altmann B, Stelljes M, Lenz G, Marx J, Hasenkamp J, et al. Results of the astral study: a prospective phase ii clinical study of the german lymphoma alliance to assess the efficacy and toxicity of high-dose chemotherapy followed by allogeneic stem cell transplantation as treatment of primary progressive and relapsed aggressive non-hodgkin lymphoma. Blood. 2023;142(1):231.

[26] Krämer I, König L, Luft T, Hegenbart U, Schönland S, Eichkorn T, et al. Intermediate-dose TBI/fludarabine conditioning for allogeneic hematopoietic cell transplantation in patients with peripheral T-cell lymphoma. Bone Marrow Transplant. 2025;60(5):581–6. 39948382 10.1038/s41409-025-02522-4 PMC12061767

[27] Berning P, Schmitz N, Ngoya M, Finel H, Boumendil A, Wang F, et al. Allogeneic hematopoietic stem cell transplantation for NK/T-cell lymphoma: an international collaborative analysis. Leukemia. 2023;37(7):1511–20. 37157017 10.1038/s41375-023-01924-x PMC10166457

[28] Karsten IE, Schmitz N, Fekom M, de Leval L, Finel H, Khvedelidze I, et al. Hematopoietic stem cell transplantation in hepatosplenic T-cell lymphoma: a retrospective analysis of the EBMT lymphoma working party. Blood. 2024;144:399. 10.1016/S2352-3026(26)00147-X 42594932

[29] Mehta-Shah N, Ito K, Bantilan K, Moskowitz AJ, Sauter C, Horwitz SM, et al. Baseline and interim functional imaging with PET effectively risk stratifies patients with peripheral T-cell lymphoma. Blood Adv. 2019;3(2):187–97. 30670535 10.1182/bloodadvances.2018024075 PMC6341183

[30] Schmitz C, Rekowski J, Müller SP, Hertenstein B, Franzius C, Ganser A, et al. Baseline and interim PET-based outcome prediction in peripheral T-cell lymphoma: A subgroup analysis of the PETAL trial. Hematol Oncol. 2020;38(3):244–56. 32067259 10.1002/hon.2697

[31] Novelli S, Bento L, Garcia I, Prieto L, López L, Gutierrez G, et al. Allogeneic stem cell transplantation in mature t cell and natural killer/T neoplasias: a registry study from spanish GETH/GELTAMO centers. Transplant and Cellular Ther. 2021;27(6):493-e1. 10.1016/j.jtct.2021.03.014 33857447

[32] Wulf G, Hasenkamp J, Jung W, Wilhelm C, Held G, Nickelsen M, et al. Allogeneic stem cell transplantation for patients with relapsed or refractory T-cell lymphoma: efficacy of lymphoma-directed conditioning against advanced disease. Bone Marrow Transplant. 2019;54(6):877–84. 30413811 10.1038/s41409-018-0360-9

