# Supplementary material for: Allogeneic stem cell transplantation for major T-cell lymphoma entities: an analysis of the EBMT-lymphoma working party
Source: J Hematol Oncol. 2026 Feb 21;19:17. doi: 10.1186/s13045-026-01783-w (PMC12930567; doi:10.1186/s13045-026-01783-w)

Supplemental Figure S1 (CR by CT vs. CMR by PET at allo-SCT)

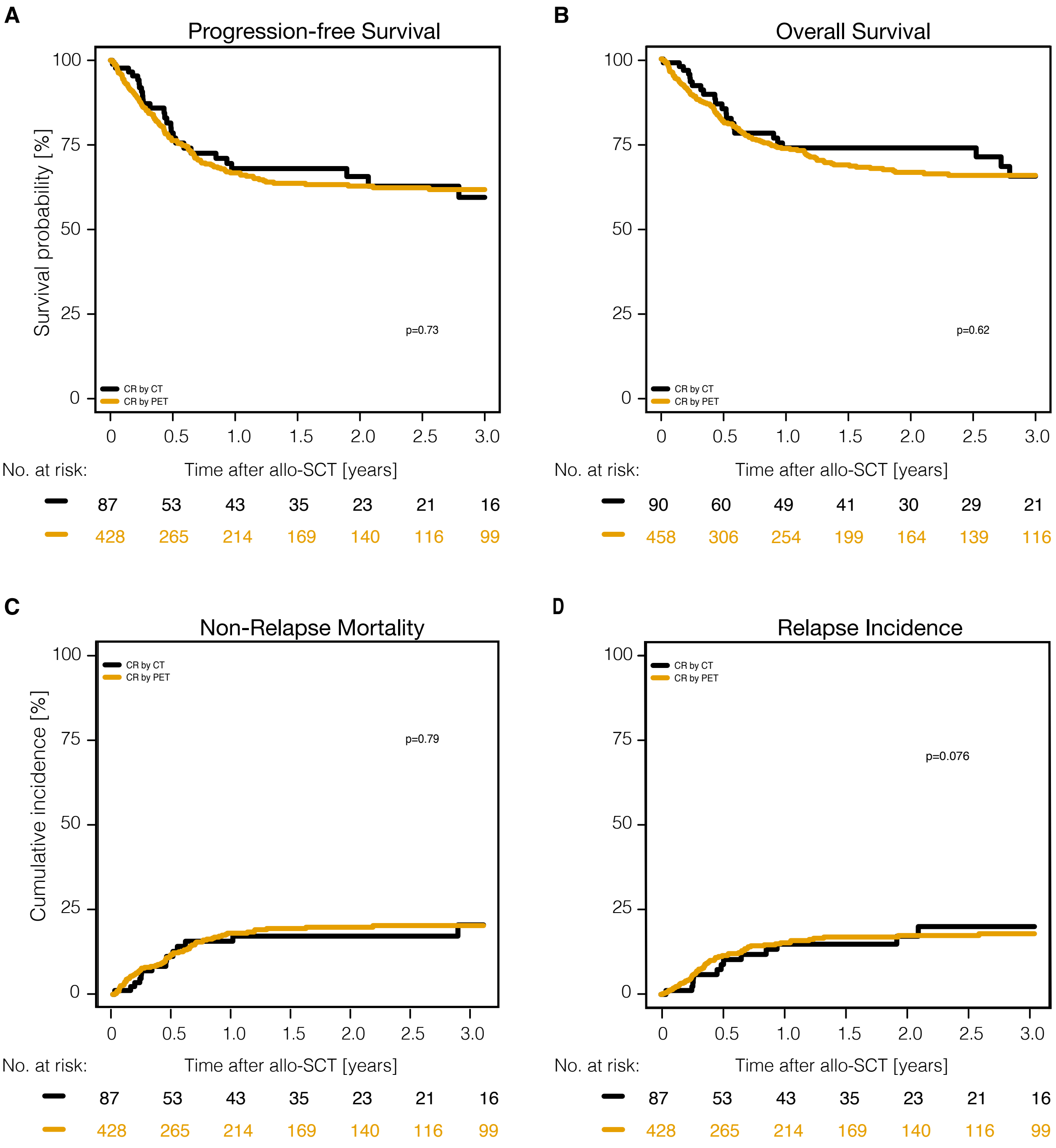

Supplemental Figure S2 (PR by CT vs. non-CMR by PET at allo-SCT)

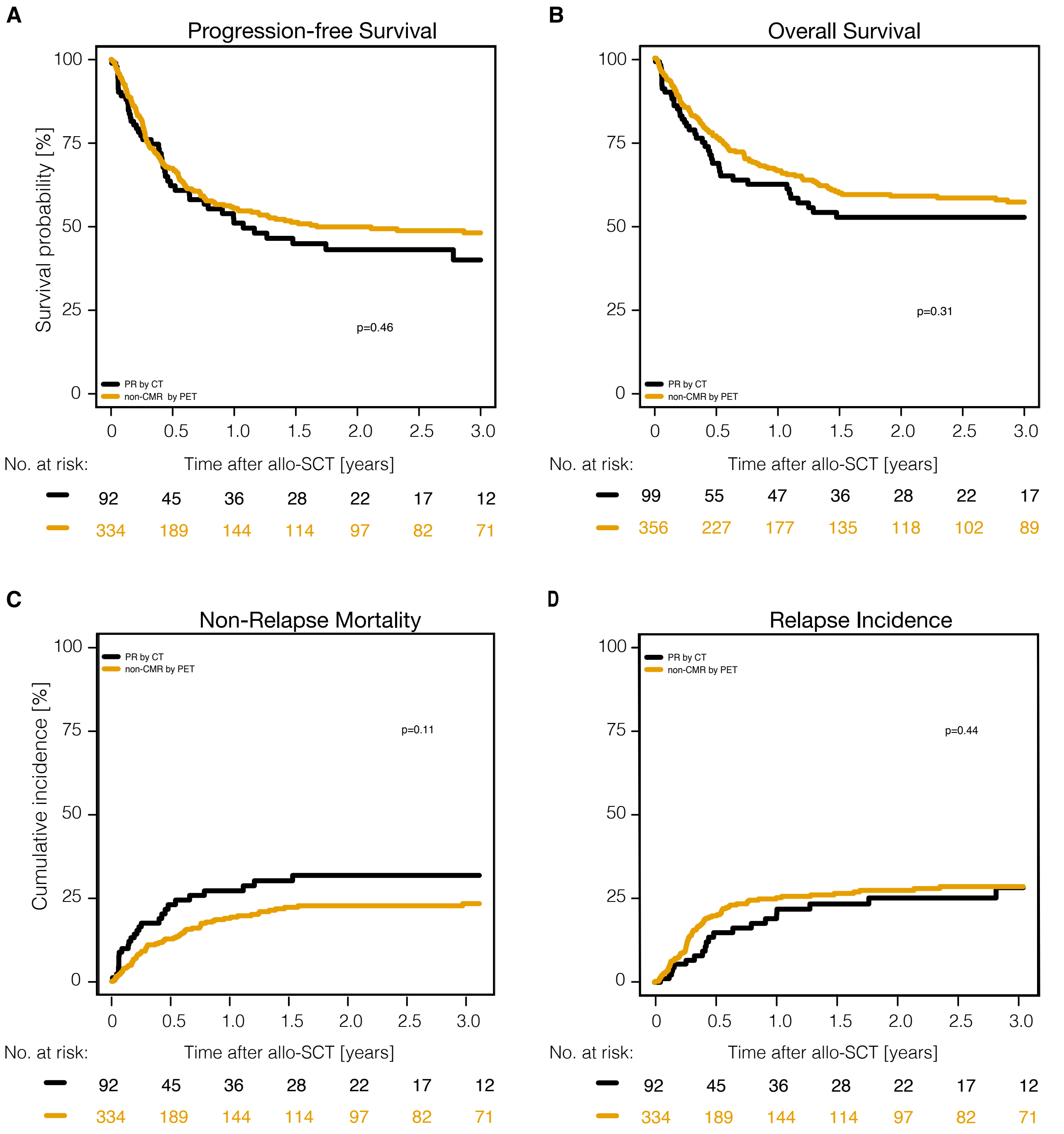

Supplemental Figure S3 (Number of therapy lines prior allo-SCT)

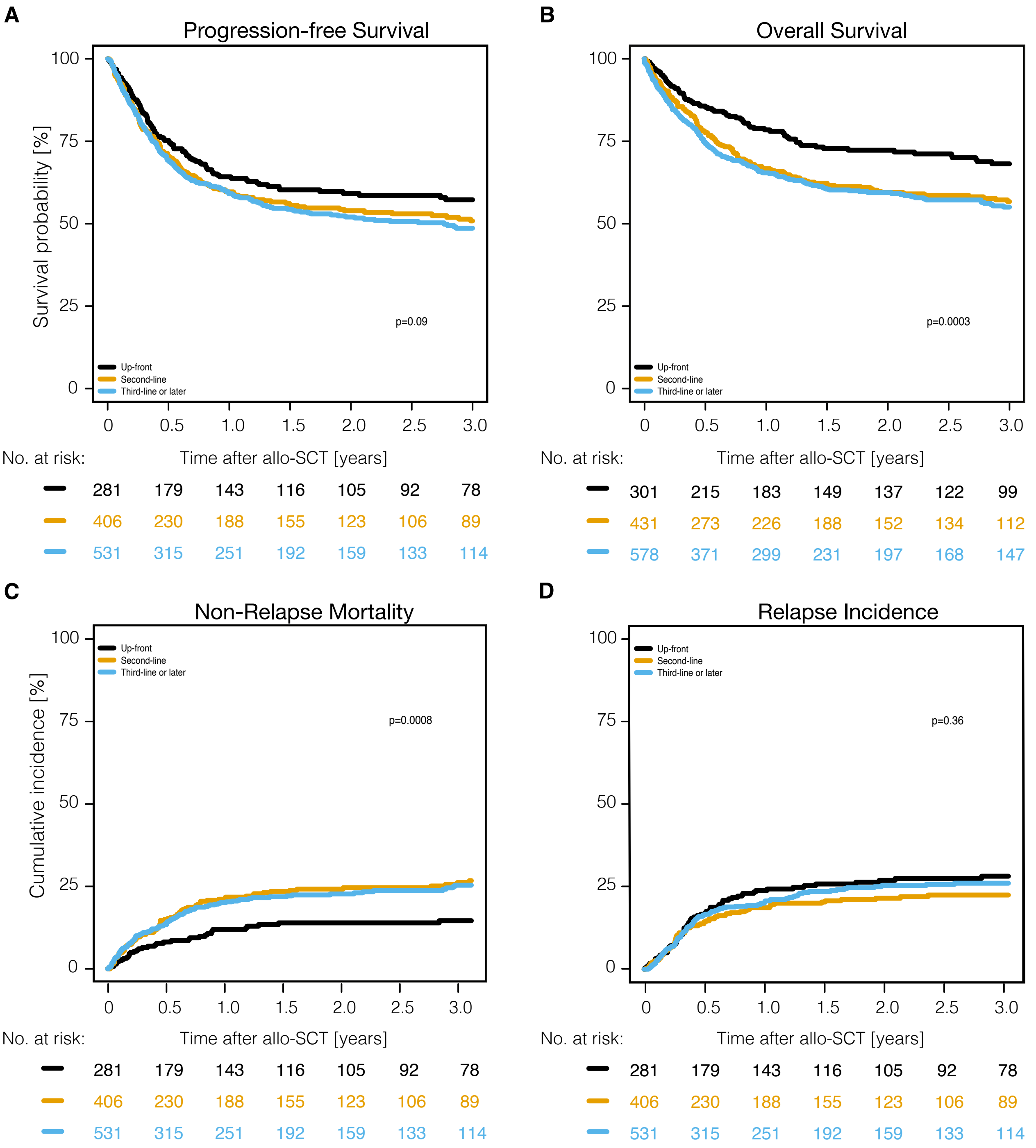

Supplemental Figure S4 (auto-SCT prior allo-SCT)

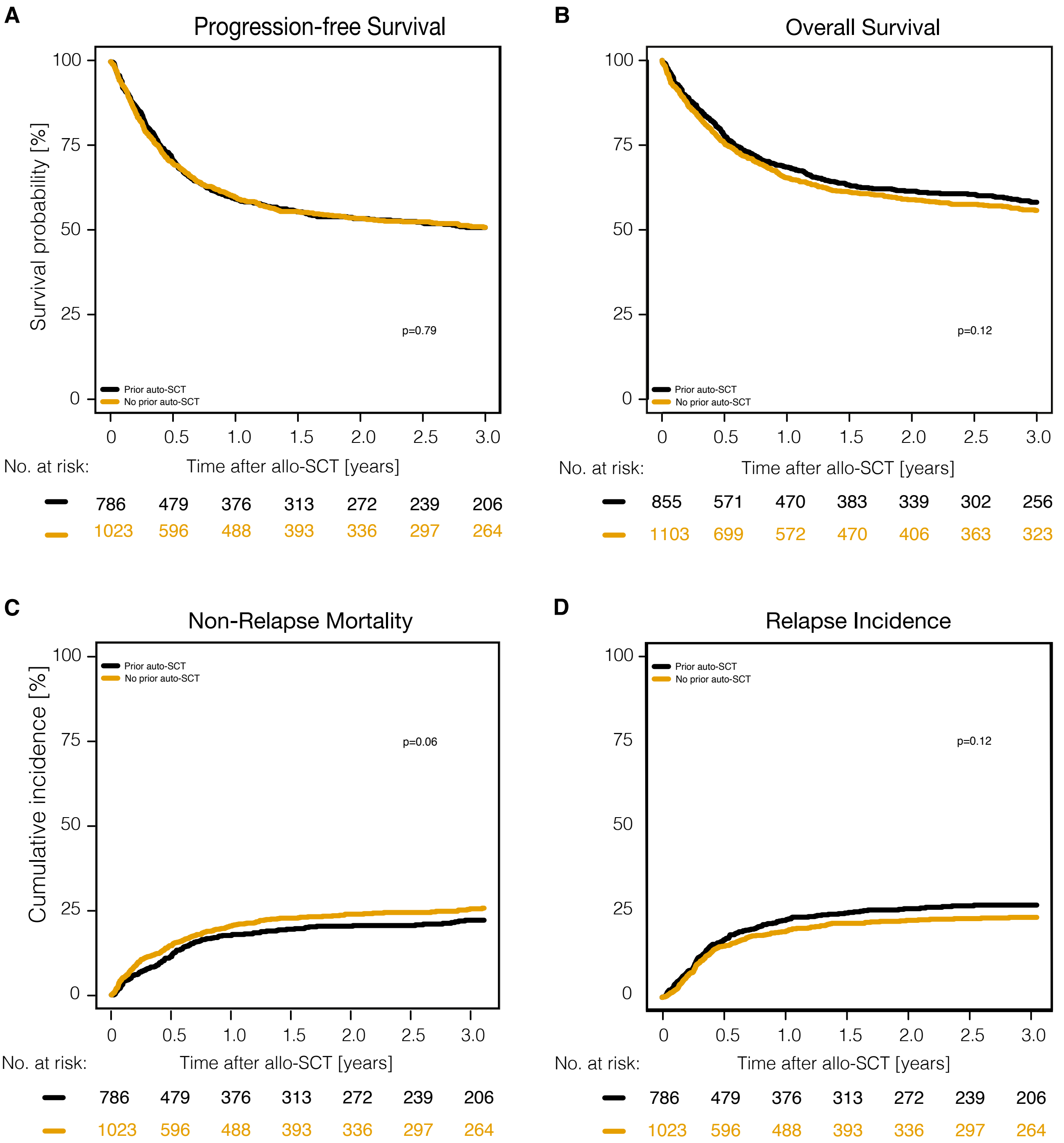

Supplemental Figure S5 (CR patients at allo-SCT)

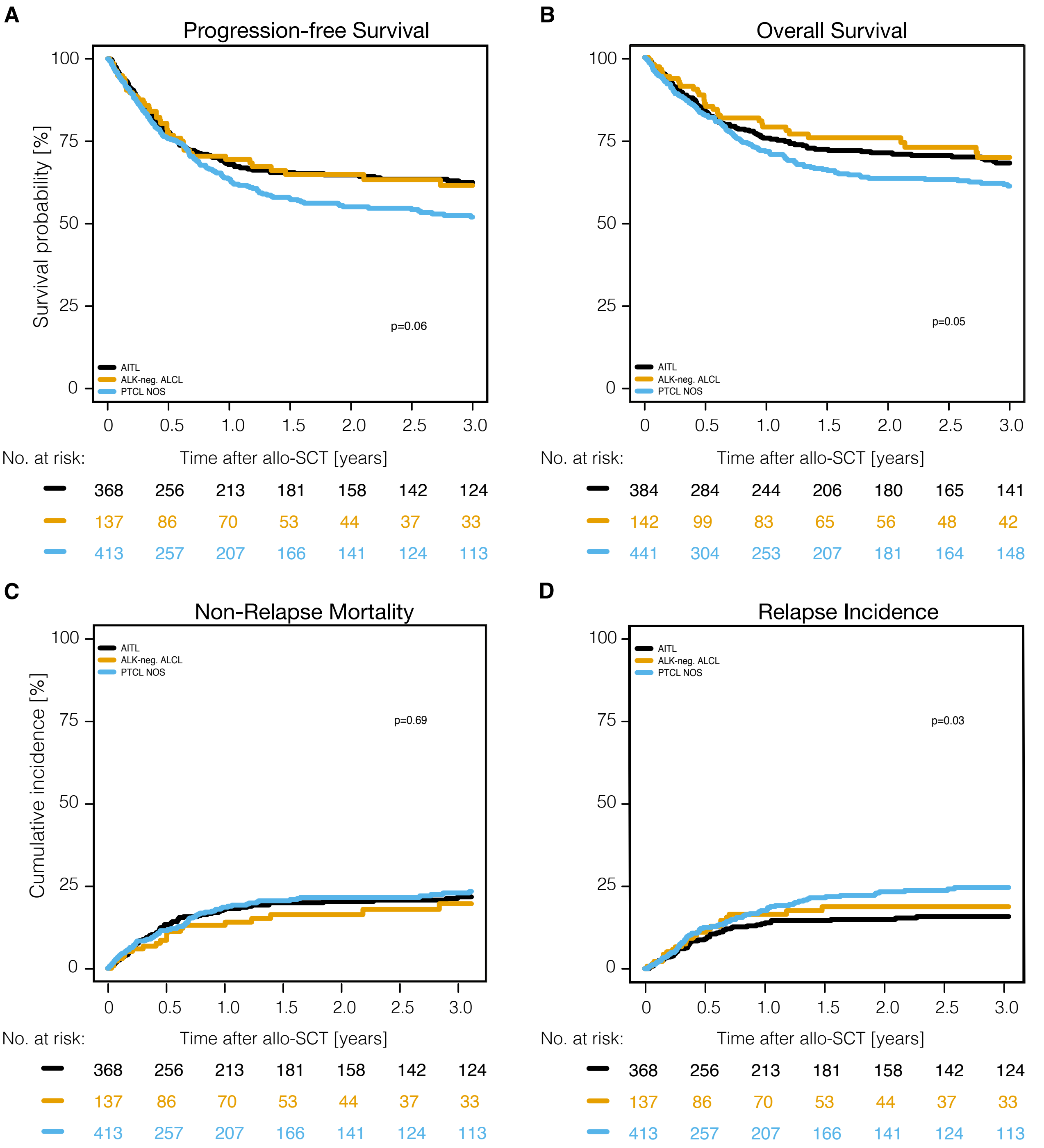

Supplemental Figure S6 (PR patients at allo-SCT)

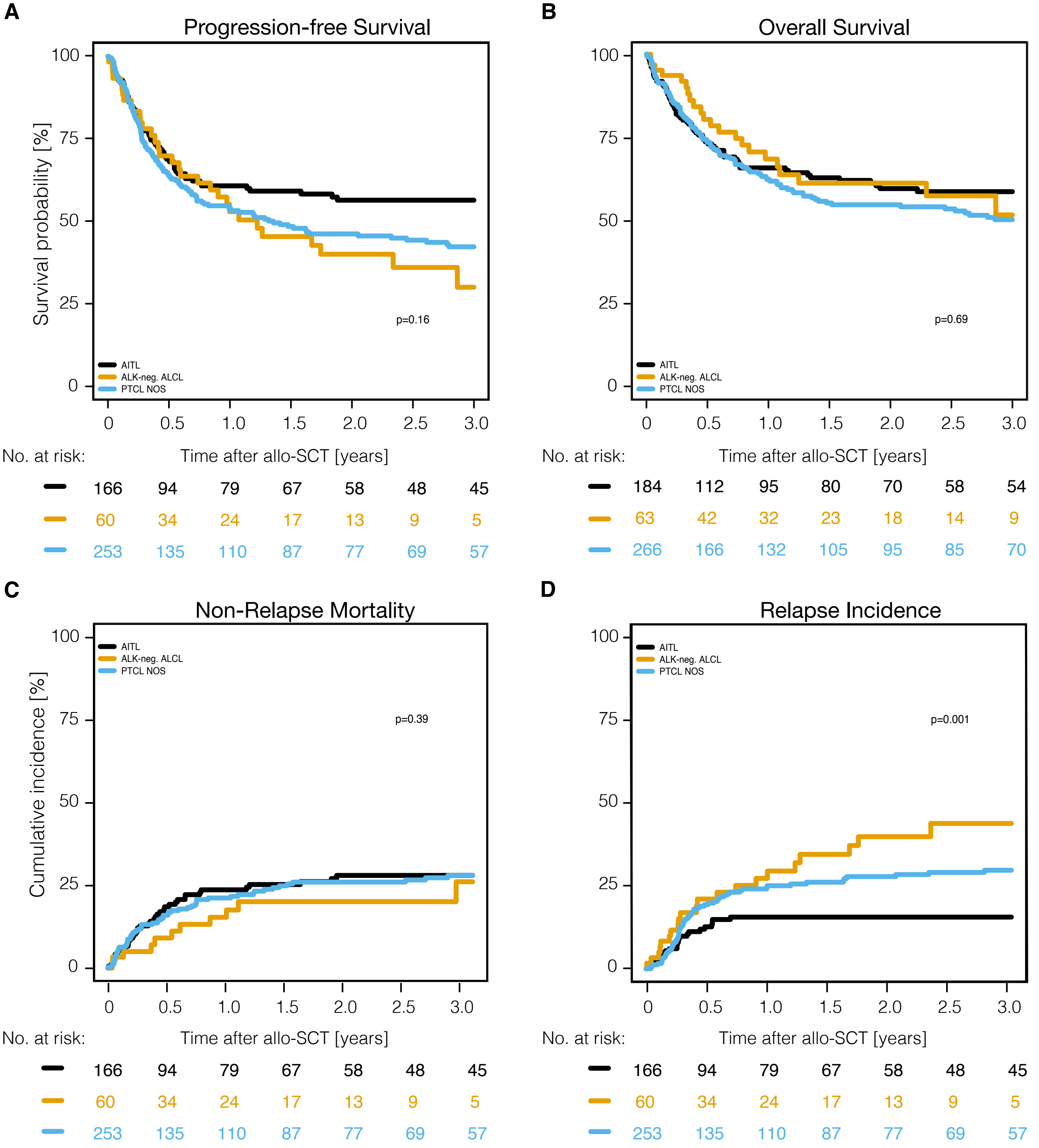

Supplemental Figure S7 (SD vs. PD at allo-SCT)

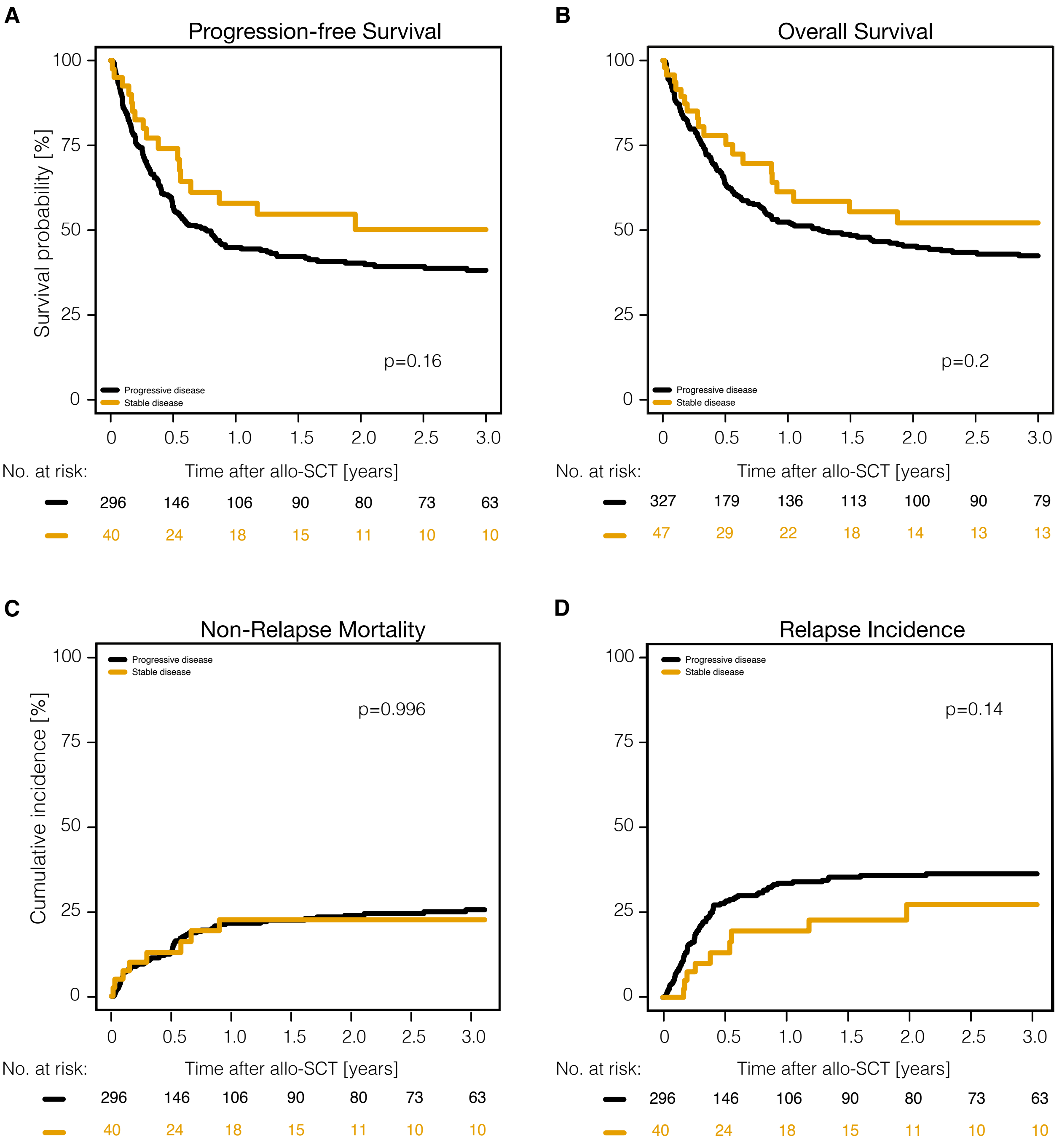

Suppl. Fig. S8 (RI after allo-SCT with PD at SCT)

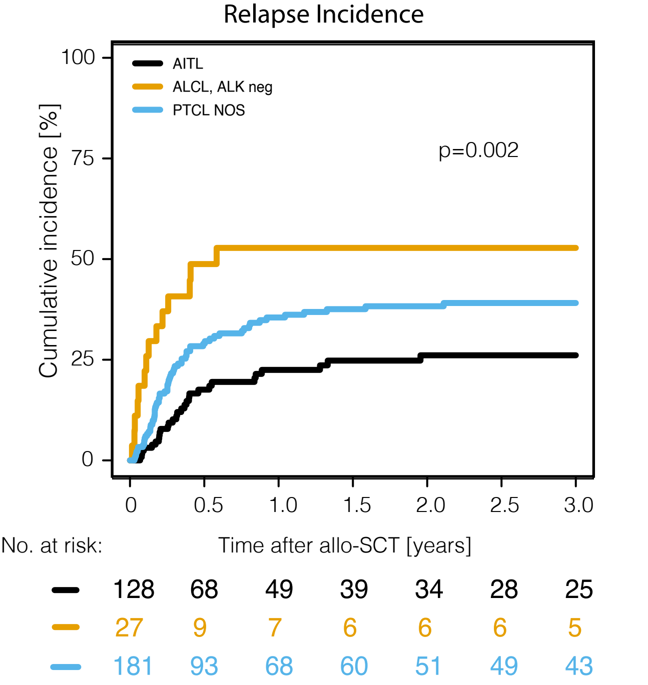

Supplement: Supplementary file 1 — Additional file 1. [file 13045_2026_1783_MOESM1_ESM.pdf]
