# Supplementary material for: Allogeneic stem cell transplantation for major T-cell lymphoma entities: an analysis of the EBMT-lymphoma working party
Source: J Hematol Oncol. 2026 Feb 21;19:17. doi: 10.1186/s13045-026-01783-w (PMC12930567; doi:10.1186/s13045-026-01783-w)
Supplement: Supplementary file 2 — Additional file 2. [file 13045_2026_1783_MOESM2_ESM.docx]

## **Supplemental Table S1. Details of the conditioning regimens used most frequently in the study.**

## TBI, total body irradiation, BuCyFlu, busulfan, fludarabine, cyclophosphamide; BuFlu, busulfan, fludarabine, BuCy, busulfan, cyclophosphamide;

## CyFlu, cyclophosphamide, fludarabine; FluMel, fludarabine, melphalan; Treo, treosulfa; RIC, reduced intensity conditioning: MAC, myeloablative conditioning.

| **Variable/Dose** | **TBI based,** | **BuCyFlu,** | **BuFlu based,** | **BuCy,** | **CyFlu,** | **FluMel based,** | **Treo based** |
| --- | --- | --- | --- | --- | --- | --- | --- |
|  | **n=499** | **n=214** | **n=502** | **n=25** | **n=145** | **n=341** | **n=89** |
| **TBI Gy** |  |  |  |  |  |  |  |
| 2 to 5 Gy | 217 (44.1) |  |  |  |  |  |  |
| 6 to 9 Gy | 125 (25.4) |  |  |  |  |  |  |
| >10 Gy | 150 (30.5) |  |  |  |  |  |  |
| Unknown | 7 |  |  |  |  |  |  |
| **Busulfan, mg/kg** |  |  |  |  |  |  |  |
| <6.4 mg/kg |  | 8 (4.7) | 42 (8.9) | 3 (13) |  |  |  |
| ≥6.4 mg/kg iv |  | 43 (25.4) | 207 (43.9) | 0 (0) |  |  |  |
| ≥6.4 mg/kg, application unknown |  | 9 (5.3) | 51 (10.8) | 0 (0) |  |  |  |
| ≥8 mg/kg |  | 109 (64.5) | 172 (36.4) | 20 (87) |  |  |  |
| Unknown |  | 45 | 30 | 2 |  |  |  |
| **Cyclophosphamide,** **mg/kg** |  |  |  |  |  |  |  |
| ≤60 mg/kg | 108 (50.9) | 57 (33.5) |  | 2 (8.3) | 76 (65.5) | 10 (76.9) |  |
| >60 mg/kg | 104 (49.1) | 113 (66.5) |  | 22 (91.7) | 40 (34.5) | 3 (23.1) |  |
| Unknown | 287 | 44 |  | 1 | 29 | 328 |  |
| **Fludarabine, mg/m^2^** |  |  |  |  |  |  |  |
| <120 mg/m^2^ | 90 (27.4) | 7 (4.1) | 16 (3.4) |  | 17 (20) | 30 (9.8) | 5 (6.4) |
| 120 to 149 mg/m^2^ | 100 (30.4) | 101 (59.4) | 57 (12.2) |  | 44 (51.8) | 45 (14.7) | 12 (15.4) |
| 150 to 200 mg/m^2^ | 135 (41) | 62 (36.5) | 387 (82.9) |  | 24 (28.2) | 220 (71.9) | 61 (78.2) |
| >200 mg/m^2^ | 4 (1.2) | 0 (0) | 7 (1.5) |  | 0 (0) | 11 (3.6) | 0 (0) |
| Unknown | 170 | 44 | 35 |  | 60 | 35 | 11 |
| **Melphalan, mg/m^2^** |  |  |  |  |  |  |  |
| <100 mg/m^2^ |  |  |  |  |  | 9 (2.9) |  |
| 100-140 mg/m^2^ |  |  |  |  |  | 301 (95.9) |  |
| 200 mg/mg^2^ |  |  |  |  |  | 4 (1.3) |  |
| Unknown |  |  |  |  |  | 27 |  |
| **Treosulfan, g/m^2^** |  |  |  |  |  |  |  |
| <24 g/m^2^ |  |  |  |  |  |  | 5 (15.6) |
| 30 g/m^2^ |  |  |  |  |  |  | 27 (84.4) |
| Unknown |  |  |  |  |  |  | 57 |
| **Conditioning regimen** |  |  |  |  |  |  |  |
| RIC | 229 (46.4) | 69 (33.2) | 354 (71.5) | 1 (4) | 100 (69) | 292 (87.4) | 47 (52.8) |
| MAC | 265 (53.6) | 139 (66.8) | 141 (28.5) | 24 (96) | 45 (31) | 42 (12.6) | 42 (47.2) |
| Unknown | 5 | 6 | 7 | 0 | 0 | 7 | 0 |

## **Supplemental Table S2.** Number of allo-SCTs in the investigated time period (2010-2022).

## Allo-SCT, allogeneic stem cell transplantation; CR, complete remission; PR, partial response; SD, stable disease, PD, progressive disease; IQR, interquartile range.

| **Variable** | **allo-SCT** |
| --- | --- |
|  | **n (%)** |
| **Year** |  |
| 2010 | 114 (5.8) |
| 2011 | 120 (6.1) |
| 2012 | 133 (6.8) |
| 2013 | 159 (8.1) |
| 2014 | 152 (7.8) |
| 2015 | 137 (7.0) |
| 2016 | 144 (7.4) |
| 2017 | 152 (7.8) |
| 2018 | 163 (8.3) |
| 2019 | 184 (9.4) |
| 2020 | 183 (9.3) |
| 2021 | 178 (9.1) |
| 2022 | 139 (7.1) |
| **Median age at allo-SCT, range [IQR]** |  |
| 2010-2012 | 51.9 (18.3-73.2) |
| 2013-2015 | 54.8 (19.9-75.9) |
| 2016-2018 | 56.2 (18.1-78.5) |
| 2019-2022 | 52.2 (18.0-74.8) |
| **Proportion of patients aged ≥ 65 years receiving allo-SCT, n (%)** |  |
| 2010-2012 | 37 (8.3%) |
| 2013-2015 | 55 (12%) |
| 2016-2018 | 110 (16.1%) |
| 2019-2022 | 27 (7.4%) |
| **Proportion of CR+PR/SD+PD patients at allo-SCT, n (%)** |  |
| 2010-2012 | 272/80 (77.3%/22.7%) |
| 2013-2015 | 302/117 (72.1%/27.9%) |
| 2016-2018 | 364/70 (83.9%/16.1%) |
| 2019-2022 | 542/107 (83.5%/16.5%) |
| **CR rate at allo-SCT, n (%)** |  |
| 2010-2012 | 178 (18.4%) |
| 2013-2015 | 196 (20.3%) |
| 2016-2018 | 236 (24.4%) |
| 2019-2022 | 357 (36.9%) |

##

## **Supplemental Table S3.** Major characteristics of patients undergoing allo-SCT after one line of therapy. Allo-SCT, allogeneic stem cell transplantation; IQR, interquartile range; ALK-negative ALCL, anaplastic lymphoma kinase-negative anaplastic large cell lymphoma; AITL, angioimmunoblastic T-cell lymphoma; PTCL NOS, peripheral T-cell lymphoma not otherwise specified; ATG, anti-thymocyte globulin;  RIC, reduced-intensity conditioning; MAC, myeloablative conditioning; PTCY, post-transplant cyclophosphamide; CT, computed tomography; PET, positron emission tomography; CR, complete remission; PR, partial remission; SD/PD, stable disease/progressive disease; IPI, international prognostic index; GvHD, graft-versus-host disease; CSA, cyclosporine; MMF, mycophenolate mofetil; MTX, methotrexate;  Results expressed as n (%) unless otherwise stated.

| **Variable** | **Total** | **PTCL NOS** | **AITL** | **ALK-neg. ALCL** | **p-value** |
| --- | --- | --- | --- | --- | --- |
|  | **n=301 (100%)** | **n=116 (38.4%)** | **n=51 (16.9%)** | **n=134 (44.5%)** |  |
| **Median age at allo-SCT (range) [IQR]** | 55.61 (18.5-74.5)  [46.6-60.5] | 56.1 (33.9-74.5) [50-60.6] | 55.8 (25.3-70.8) [46.8-59.1] | 53.2 (18.5-70.9) [43.1-60.6] | 0.0883 |
| **Sex patient** |  |  |  |  | 0.3369 |
| Female | 99 (32.9) | 44 (37.9) | 15 (29.4) | 40 (29.9) |  |
| Male | 202 (67.1) | 72 (62.1) | 36 (70.6) | 94 (70.1) |  |
| **Sex donor** |  |  |  |  | 0.9341 |
| Female | 99 (33.1) | 39 (34.2) | 16 (31.4) | 44 (32.8) |  |
| Male | 200 (66.9) | 75 (65.8) | 35 (68.6) | 90 (67.2) |  |
| Unknown | 2 | 2 | 0 | 0 |  |
| **Female to male donor combination** |  |  |  |  | 0.9685 |
| No | 243 (81.3) | 92 (80.7) | 42 (82.4) | 109 (81.3) |  |
| Yes | 56 (18.7) | 22 (19.3) | 9 (17.6) | 25 (18.7) |  |
| Unknown | 2 | 2 | 0 | 0 |  |
| **Type of donor** |  |  |  |  |  |
| Haploidentical | 31 (10.3) | 9 (7.8) | 9 (17.6) | 13 (9.7) | 0.2746 |
| Matched related donor | 114 (37.9) | 45 (38.8) | 21 (41.2) | 48 (35.8) |  |
| Unrelated donor | 156 (51.8) | 62 (53.4) | 21 (41.2) | 73 (54.5) |  |
| **CMV status/patient** |  |  |  |  | 0.1151 |
| Negative | 128 (42.7) | 50 (43.1) | 15 (30) | 63 (47) |  |
| Positive | 172 (57.3) | 66 (56.9) | 35 (70) | 71 (53) |  |
| Unknown | 1 | 0 | 1 | 0 |  |
| **CMV status/donor** |  |  |  |  | 0.2917 |
| Negative | 160 (54.1) | 65 (56.5) | 22 (44) | 73 (55.7) |  |
| Positive | 136 (45.9) | 50 (43.5) | 28 (56) | 58 (44.3) |  |
| Unknown | 5 | 1 | 1 | 3 |  |
| **CMV donor to patient** |  |  |  |  |  |
| Negative to negative | 98 (33.2) | 37 (32.2) | 12 (24.5) | 49 (37.4) |  |
| Negative to positive | 62 (21) | 28 (24.3) | 10 (20.4) | 24 (18.3) |  |
| Positive to negative | 28 (9.5) | 13 (11.3) | 3 (6.1) | 12 (9.2) |  |
| Positive to positive | 107 (36.3) | 37 (32.2) | 24 (49) | 46 (35.1) |  |
| Unknown | 6 | 1 | 2 | 3 |  |
| ***In vivo* T-cell depletion** |  |  |  |  | 0.108 |
| No | 104 (35.1) | 36 (31.6) | 24 (48) | 44 (33.3) |  |
| Yes | 192 (64.9) | 78 (68.4) | 26 (52) | 88 (66.7) |  |
| Unknown | 5 | 2 | 1 | 2 |  |
| ***In vivo* T-cell depletion** |  |  |  |  |  |
| ATG | 144 (48.6) | 56 (49.1) | 19 (38) | 69 (52.3) | 0.28 |
| ATG + Campath | 1 (0.3) | 0 (0) | 0 (0) | 1 (0.8) |  |
| Campath | 47 (15.9) | 22 (19.3) | 7 (14) | 18 (13.6) |  |
| No T-cell depletion | 104 (35.1) | 36 (31.6) | 24 (48) | 44 (33.3) |  |
| Unknown | 5 | 2 | 1 | 2 |  |
| **Conditioning regimen** |  |  |  |  | 0.3204 |
| RIC | 190 (64) | 76 (65.5) | 35 (71.4) | 79 (59.8) |  |
| MAC | 107 (36) | 40 (34.5) | 14 (28.6) | 53 (40.2) |  |
| missing | 4 | 0 | 2 | 2 |  |
| **PTCY** |  |  |  |  | 0.3888 |
| No | 238 (81) | 93 (82.3) | 37 (74) | 108 (82.4) |  |
| Yes | 56 (19) | 20 (17.7) | 13 (26) | 23 (17.6) |  |
| Unknown | 7 | 3 | 1 | 3 |  |
| **Ann Arbor stage at diagnosis** |  |  |  |  | 0.02 |
| I-II | 8 (8.2) | 1 (2.3) | 2 (33.3) | 5 (10.6) |  |
| III | 27 (27.8) | 14 (31.8) | 3 (50) | 10 (21.3) |  |
| IV | 62 (63.9) | 29 (65.9) | 1 (16.7) | 32 (68.1) |  |
| Unknown | 204 | 72 | 45 | 87 |  |
| **Performed CT scan at allo-SCT** |  |  |  |  | 0.9167 |
| No | 35 (16.1) | 13 (15.5) | 8 (18.2) | 14 (15.7) |  |
| Yes | 182 (83.9) | 71 (84.5) | 36 (81.8) | 75 (84.3) |  |
| Unknown | 84 | 32 | 7 | 45 |  |
| **Performed PET scan at allo-SCT** |  |  |  |  | 0.6448 |
| Negative | 105 (66.9) | 38 (65.5) | 25 (73.5) | 42 (64.6) |  |
| Positive | 52 (33.1) | 20 (34.5) | 9 (26.5) | 23 (35.4) |  |
| missing | 144 | 58 | 17 | 69 |  |
| **Number of lines prior to allo-SCT** |  |  |  |  | Not done |
| 1 | 301 (100) | 116 (100) | 51 (100) | 134 (100) |  |
| **Disease status at allo-SCT** |  |  |  |  | 0.24 |
| CR | 191 (65.6) | 75 (67) | 36 (75) | 80 (61.1) |  |
| PR | 74 (25.4) | 25 (22.3) | 11 (22.9) | 38 (29) |  |
| Progressive or stable disease | 26 (8.9) | 12 (10.7) | 1 (2.1) | 13 (9.9) |  |
| Unknown | 10 | 4 | 3 | 3 |  |
| **Disease status at HSCT** |  |  |  |  |  |
| CR/PR +1 line | 265 (91.1) | 100 (89.3) | 47 (97.9) | 118 (90.1) | 0.37 |
| PD | 15 (5.2) | 8 (7.1) | 0 (0) | 7 (5.3) |  |
| SD | 11 (3.8) | 4 (3.6) | 1 (2.1) | 6 (4.6) |  |
| missing | 10 | 4 | 3 | 3 |  |
| **International Prognostic Index at first diagnosis** |  |  |  |  | 0.1 |
| Low risk (0-1 score points) | 13 (10.7) | 3 (6.2) | 6 (20) | 4 (9.1) |  |
| Low-intermediate risk (2) | 27 (22.1) | 9 (18.8) | 11 (36.7) | 7 (15.9) |  |
| High-intermediate risk (3) | 53 (43.4) | 24 (50) | 9 (30) | 20 (45.5) |  |
| High risk (4 or 5) | 29 (23.8) | 12 (25) | 4 (13.3) | 13 (29.5) |  |
| Unknown | 179 | 68 | 21 | 90 |  |
| **Karnofsky Index at allo-SCT** |  |  |  |  | 0.935 |
| < 90 | 75 (26.1) | 30 (27.3) | 12 (26.1) | 33 (25.2) |  |
| >= 90 | 212 (73.9) | 80 (72.7) | 34 (73.9) | 98 (74.8) |  |
| Unknown | 14 | 6 | 5 | 3 |  |
| **HCT-CI index** |  |  |  |  | 0.34 |
| 0 | 127 (52.3) | 48 (52.7) | 18 (43.9) | 61 (55) |  |
| 1 to 2 | 58 (23.9) | 20 (22) | 15 (36.6) | 23 (20.7) |  |
| 3+ | 58 (23.9) | 23 (25.3) | 8 (19.5) | 27 (24.3) |  |
| Unknown | 58 | 25 | 10 | 23 |  |
| **GvHD prophylaxis** |  |  |  |  | Not done |
| CSA based | 64 (21.6) | 26 (22.8) | 13 (26) | 25 (18.9) |  |
| CSA MMF based | 63 (21.3) | 27 (23.7) | 5 (10) | 31 (23.5) |  |
| CSA MTX/ MMF+MTX based | 76 (25.7) | 26 (22.8) | 12 (24) | 38 (28.8) |  |
| MMF/ MTX based | 27 (9.1) | 10 (8.8) | 6 (12) | 11 (8.3) |  |
| Other | 10 (3.4) | 5 (4.4) | 1 (2) | 4 (3) |  |
| PTCY based | 56 (18.9) | 20 (17.5) | 13 (26) | 23 (17.4) |  |
| Unknown | 5 | 2 | 1 | 2 |  |

## **Supplemental Table S4.** Characteristics of patients undergoing allo-SCT with stable or progressive disease.

## Allo-SCT, allogeneic stem cell transplantation; SD, stable disease; PD, progressive disease; PR, partial response; CR, complete response.

## **A)** Availability of CT and PET data at the time point of entering allo-SCT among patients with stable and progressive disease at allo-SCT. CT, computed tomography; PET, positron-emission tomography.

| **Parameter** | **SD/PD** | **progressive disease** | **stable disease** |
| --- | --- | --- | --- |
|  | **n=374** | **n=327** | **n=47** |
| **CT scan** |  |  |  |
| No | 19 (11.7) | 14 (11.4) | 5 (12.8) |
| Yes | 143 (88.3) | 109 (88.6) | 34 (87.2) |
| Unknown | 212 | 204 | 8 |
| **PET status** |  |  |  |
| Negative | 8 (7.2) | 3 (3.4) | 5 (20.8) |
| Positive | 103 (92.8) | 84 (96.6) | 19 (79.2) |
| Unknown | 263 | 240 | 23 |

## **B)** 3-year outcomes of allo-SCT depending on remission status at allo-SCT.

## Allo-SCT, allogeneic stem cell transplantation; CR, complete response; PR, partial response; SD, stable disease; PD, progressive disease; OS, overall survival; PFS, progression-free survival; RI, relapse incidence; NRM, non-relapse mortality.

|  | **CR** | **PR** | **SD** | **PD** | **P value** |
| --- | --- | --- | --- | --- | --- |
| **OS** | 65.2% [61.7-68.5] | 53.7% [48.6-58.4] | 52.2% [35.1-66.7] | 42.4% [36.5-48.3] | <0.0001 |
| **PFS** | 57.8% [54.2-61.3] | 46% [40.9-51] | 50.2% [31.7-66.1] | 38.2% [32.1-44.2] | <0.0001 |
| **RI** | 20.2% [17.4-23.1] | 26.5% [22.3-30.9] | 27.3% [12.9-44] | 36.4% [30.6-42.2] | <0.0001 |
| **NRM** | 22% [19.1-25] | 27.5% [23.2-32] | 22.5% [10.3-37.7] | 25.4% [20.3-30.9] | 0.0535 |

## **C)** 5-year outcomes of allo-SCT among patients with SD/PD at allo-SCT.

Allo-SCT, allogeneic stem cell transplantation; SD, stable disease; PD, progressive disease; OS, overall survival; PFS, progression-free survival; RI, relapse incidence; NRM, non-relapse mortality.

| **5-year outcomes post-allo-SCT** | **N** | **Event (N)** | **Estimation (IC95%)** |
| --- | --- | --- | --- |
| OS | 374 | 196 | 38.1 (32.3 - 43.9) |
| PFS | 336 | 189 | 36.6 (30.8 - 42.4) |
| RI | 336 | 110 | 35.9 (30.4 - 41.4) |
| NRM | 336 | 79 | 27.5 (22.3 - 33) |

## **Supplemental Table S5A.** Multivariate Cox proportional-hazards models of outcomes of allo-SCT for all patients (ALK-neg. ALCL, PTCL NOS, AITL). allo-SCT, allogeneic stem cell transplantation; HR=hazard ratio; CI=confidence interval; ALK-neg. ALCL, anaplastic lymphoma kinase-negative anaplastic large cell lymphoma; AITL, angioimmunoblastic T-cell lymphoma; PTCL NOS, peripheral T-cell lymphoma not otherwise specified; MRD, matched related donor; UD, unrelated donor; CR, complete remission; PR, partial remission; Referent groups for Cox proportional-hazards analysis are highlighted in **bold**.

|  | | **Progression-free Survival** | | **Overall Survival** | | **Relapse Incidence** | | **Non-relapse Mortality** | |
| --- | --- | --- | --- | --- | --- | --- | --- | --- | --- |
| **Variable** | | **HR (95% CI)** | **P-value** | **HR (95% CI)** | **P-value** | **HR (95% CI)** | **P-value** | **HR (95% CI)** | **P-value** |
| Lymphoma | **AITL** | 1 |  | 1 |  | 1 |  | 1 |  |
|  | ALK-neg. ALCL | 1.36 (1.03-1.8) | **0.03** | 1.23 (0.9-1.67) | 0.19 | 1.76 (1.18-2.61) | **0.005** | 1.1 (0.74-1.63) | 0.65 |
|  | PTCL NOS | 1.38 (1.12-1.7) | **0.002** | 1.39 (1.12-1.73) | **0.003** | 1.75 (1.29-2.38) | **<0.001** | 1.13 (0.85-1.51) | 0.4 |
| Year of allo-SCT | **2010 to 2015** | 1 |  | 1 |  | 1 |  | 1 |  |
|  | 2016 to 2022 | 0.94 (0.75-1.17) | 0.56 | 1.18 (0.92-1.52) | 0.19 | 0.84 (0.63-1.12) | 0.24 | 1.11 (0.8-1.55) | 0.53 |
| Age at allo-SCT  (by ten year increments) | | 1.14 (1.04-1.24) | **0.005** | 1.28 (1.16-1.41) | **<0.001** | 0.97 (0.87-1.09) | 0.6 | 1.37 (1.19-1.57) | **<0.001** |
| Type of donor | **Haploidentical** | 1 |  | 1 |  | 1 |  | 1 |  |
|  | MRD | 0.84 (0.62-1.13) | 0.25 | 0.67 (0.49-0.92) | **0.01** | 1.32 (0.82-2.13) | 0.25 | 0.60 (0.40-0.90) | **0.01** |
|  | UD | 0.8 (0.59-1.07) | 0.14 | 0.66 (0.48-0.9) | **0.01** | 1.1 (0.68-1.76) | 0.71 | 0.66 (0.45-0.97) | **0.04** |
| Female to male donor | **No** | 1 |  | 1 |  | 1 |  | 1 |  |
|  | Yes | 1.19 (0.95-1.5) | 0.13 | 1.22 (0.96-1.56) | 0.11 | 1 (0.72-1.39) | 0.99 | 1.41 (1.03-1.93) | **0.03** |
| Karnofsky index | **< 90** | 1 |  | 1 |  | 1 |  | 1 |  |
|  | >= 90 | 0.85 (0.7-1.03) | 0.09 | 0.78 (0.63-0.96) | **0.02** | 0.86 (0.65-1.14) | 0.3 | 0.84 (0.64-1.11) | 0.21 |
| Number of lines prior allo-SCT | **1** | 1 |  | 1 |  | 1 |  | 1 |  |
|  | 2 | 1.12 (0.87-1.45) | 0.38 | 1.30 (0.98-1.72) | 0.07 | 0.78 (0.55-1.11) | 0.17 | 1.65 (1.12-2.45) | **0.01** |
|  | 3 or more | 1.15 (0.9-1.48) | 0.25 | 1.31 (1-1.72) | 0.053 | 0.9 (0.65-1.25) | 0.52 | 1.58 (1.07-2.32) | **0.02** |
| Disease status at allo-SCT | **CR** | 1 |  | 1 |  | 1 |  | 1 |  |
|  | PR | 1.37 (1.11-1.71) | **0.004** | 1.30 (1.03-1.65) | **0.03** | 1.65 (1.22-2.25) | **<0.001** | 1.13 (0.83-1.54) | 0.43 |
|  | Progressive or stable disease | 1.66 (1.3-2.13) | **<0.001** | 1.84 (1.42-2.39) | **<0.001** | 2.25 (1.6-3.16) | **<0.001** | 1.22 (0.85-1.76) | 0.28 |
| *In vivo* T cell depletion | **No** | 1 |  | 1 |  | 1 |  | 1 |  |
|  | Yes | 1.2 (0.98-1.48) | 0.09 | 1.19 (0.95-1.5) | 0.13 | 1.31 (0.98-1.75) | 0.07 | 1.11 (0.83-1.5) | 0.48 |
| Myeloablative regimen | **No** | 1 |  | 1 |  | 1 |  | 1 |  |
|  | Yes | 1.18 (0.97-1.44) | 0.09 | 1.2 (0.96-1.48) | 0.1 | 1.11 (0.85-1.46) | 0.45 | 1.26 (0.95-1.67) | 0.11 |

## **Supplemental Table S5B.** Multivariate Cox proportional hazards models of outcomes of allo-SCT for all patients (ALK-neg. ALCL, PTCL NOS, AITL). allo-SCT, allogeneic stem cell transplantation; aGvHD, acute graft-versus-host disease; cGvHD, chronic graft-versus-host disease; HR=hazard ratio; CI=confidence interval; ALK-neg. ALCL, anaplastic lymphoma kinase-negative anaplastic large cell lymphoma; AITL, angioimmunoblastic T-cell lymphoma; PTCL NOS, peripheral T-cell lymphoma not otherwise specified; MRD, matched-related donor; UD, unrelated donor; CR, complete remission; PR, partial remission; Referent groups for Cox proportional-hazards analysis are highlighted in **bold**.

|  | | **aGVHD I-II** | | **aGVHD III-IV** | | **cGVHD** | | **cGVHD EXTENSIVE** | |
| --- | --- | --- | --- | --- | --- | --- | --- | --- | --- |
| **Variable** | | **HR (95% CI)** | **P-value** | **HR (95% CI)** | **P-value** | **HR (95% CI)** | **P-value** | **HR (95% CI)** | **P-value** |
| Lymphoma | **AITL** | 1 |  | 1 |  | 1 |  | 1 |  |
|  | ALK-neg. ALCL | 1.05 (0.76-1.45) | 0.77 | 1.08 (0.65-1.8) | 0.77 | 0.93 (0.65-1.31) | 0.66 | 0.98 (0.6-1.58) | 0.93 |
|  | PTCL NOS | 0.74 (0.57-0.96) | **0.02** | 0.66 (0.43-1) | 0.05 | 0.72 (0.55-0.94) | **0.01** | 0.79 (0.55-1.14) | 0.21 |
| Year of allo-SCT | **2010 to 2015** | 1 |  | 1 |  | 1 |  | 1 |  |
|  | 2016 to 2022 | 0.77 (0.58-1.03) | 0.08 | 0.62 (0.4-0.96) | **0.03** | 0.76 (0.57-1.02) | 0.07 | 0.87 (0.58-1.3) | 0.49 |
| Age at allo-SCT  (by ten year increments) | | 1.06 (0.95-1.18) | 1.05 (0.95-1.18) | 0.34 | 0.96 (0.81-1.15) | 0.68 | 1.05 (0.94-1.18) | 0.38 | 1.07 (0.92-1.25) |
| Type of donor | **Haploidentical** | 1 |  | 1 |  | 1 |  | 1 |  |
|  | MRD | 0.65 (0.46-0.93) | **0.02** | 0.65 (0.38-1.13) | 0.13 | 1.5 (1-2.26) | 0.053 | 1.37 (0.79-2.37) | 0.27 |
|  | UD | 0.61 (0.43-0.87) | **0.007** | 0.63 (0.37-1.09) | 0.1 | 1.7 (1.12-2.58) | **0.01** | 1.47 (0.84-2.59) | 0.18 |
| Female to male donor | **No** | 1 |  | 1 |  | 1 |  | 1 |  |
|  | Yes | 0.81 (0.6-1.1) | 0.19 | 0.89 (0.54-1.45) | 0.63 | 1.38 (1.02-1.86) | **0.03** | 1.5 (1.01-2.23) | 0.04 |
| Karnofsky index | **< 90** | 1 |  | 1 |  | 1 |  | 1 |  |
|  | >= 90 | 1.07 (0.83-1.39) | 0.59 | 1.27 (0.82-1.95) | 0.28 | 0.92 (0.7-1.2) | 0.53 | 0.93 (0.64-1.34) | 0.69 |
| Number of lines | **1** | 1 |  | 1 |  | 1 |  | 1 |  |
|  | 2 | 0.84 (0.61-1.15) | 0.27 | 0.76 (0.46-1.28) | 0.3 | 1.2 (0.87-1.67) | 0.27 | 1.16 (0.73-1.85) | 0.54 |
|  | 3 or more | 0.93 (0.69-1.26) | 0.64 | 0.95 (0.59-1.53) | 0.82 | 1.18 (0.86-1.63) | 0.3 | 1.26 (0.8-1.97) | 0.32 |
| Disease status | **CR** | 1 |  | 1 |  | 1 |  | 1 |  |
|  | PR | 1.18 (0.89-1.55) | 0.25 | 1.14 (0.72-1.79) | 0.58 | 1.04 (0.78-1.39) | 0.78 | 1.4 (0.95-2.07) | 0.09 |
|  | Progressive or stable disease | 1.62 (1.18-2.22) | **0.003** | 1.61 (0.98-2.64) | 0.06 | 1.01 (0.71-1.46) | 0.94 | 1.27 (0.79-2.04) | 0.32 |
| *In vivo* T cell depletion | **No** | 1 |  | 1 |  | 1 |  | 1 |  |
|  | Yes | 0.78 (0.6-1.02) | 0.07 | 0.67 (0.44-1.01) | 0.058 | 0.66 (0.5-0.87) | **0.003** | 0.58 (0.4-0.85) | **0.005** |
| Myeloablative regimen | **No** | 1 |  | 1 |  | 1 |  | 1 |  |
|  | Yes | 0.96 (0.74-1.25) | 0.77 | 0.87 (0.58-1.32) | 0.52 | 1.06 (0.81-1.38) | 0.69 | 1.14 (0.79-1.64) | 0.5 |
