# Supplementary material for: Allogeneic stem cell transplantation for major T-cell lymphoma entities: an analysis of the EBMT-lymphoma working party
Source: J Hematol Oncol. 2026 Feb 21;19:17. doi: 10.1186/s13045-026-01783-w (PMC12930567; doi:10.1186/s13045-026-01783-w)
Supplement: Supplementary file 3 — Additional file 3. [file 13045_2026_1783_MOESM3_ESM.docx]

**Supplemental Figure S1.** Outcomes of allo-SCT according to CR status determined by CT and PET (CR by CT vs. CMR by PET). Allo-SCT, allogeneic stem cell transplantation; CR, complete remission; CT, computed tomography; PET, positron emission tomography; CMR, complete metabolic remission.

**Supplemental Figure S2.** Outcomes of allo-SCT according to non-CR status determined by CT and PET (PR by CT vs. non-CMR by PET). Allo-SCT, allogeneic stem cell transplantation; CR, complete remission; PR, partial remission; CT, computed tomography; CMR, complete metabolic remission; PET, positron emission tomography.\

**Supplemental Figure S3.** Outcomes of allo-SCT according to the number of therapies prior allo-SCT. Allo-SCT, allogeneic stem cell transplantation.

**Supplemental Figure S4.** Outcomes of allo-SCT according to the auto-SCT status prior. Allo-SCT, allogeneic stem cell transplantation; auto-SCT, autologous stem cell transplantation.

**Supplemental Figure S5.** Outcomes of allo-SCT according to histology among CR patients at SCT. Allo-SCT, allogeneic stem cell transplantation; CR, complete remission; ALK-negative ALCL, anaplastic lymphoma kinase-negative anaplastic large cell lymphoma; PTCL NOS, peripheral T-cell lymphoma not otherwise specified; AITL, angioimmunoblastic T-cell lymphoma.**Supplemental Figure S6.** Outcomes of allo-SCT according to histology among PR patients at SCT. Allo-SCT, allogeneic stem cell transplantation; PR, partial remission; ALK-negative ALCL, anaplastic lymphoma kinase-negative anaplastic large cell lymphoma; PTCL NOS, peripheral T-cell lymphoma not otherwise specified; AITL, angioimmunoblastic T-cell lymphoma.

**Supplemental Figure S7.** Outcomes of allo-SCT according to SD or PD status at allo-SCT. Allo-SCT, allogeneic stem cell transplantation; SD, stable disease; PD, progressive disease.

**Supplemental Figure S8.** Relapse incidence among patients undergoing SCT in progressive disease. Allo-SCT, allogeneic stem cell transplantation; PD, progressive disease. ALK-negative ALCL, anaplastic lymphoma kinase-negative anaplastic large cell lymphoma; PTCL NOS, peripheral T-cell lymphoma not otherwise specified; AITL, angioimmunoblastic T-cell lymphoma; RI, relapse incidence
